# Variation in Molybdenum Content Across Broadly Distributed Populations of *Arabidopsis thaliana* Is Controlled by a Mitochondrial Molybdenum Transporter (*MOT1*)

**DOI:** 10.1371/journal.pgen.1000004

**Published:** 2008-02-29

**Authors:** Ivan Baxter, Balasubramaniam Muthukumar, Hyeong Cheol Park, Peter Buchner, Brett Lahner, John Danku, Keyan Zhao, Joohyun Lee, Malcolm J. Hawkesford, Mary Lou Guerinot, David E. Salt

**Affiliations:** 1 Bindley Bioscience Center, Purdue University, West Lafayette, Indiana, United States of America; 2 Horticulture and Landscape Architecture, Purdue University, West Lafayette, Indiana, United States of America; 3 Rothamsted Research, Harpenden, Hertfordshire, United Kingdom; 4 Molecular and Computational Biology, University of Southern California, Los Angeles, California, United States of America; 5 Biological Sciences, Dartmouth College, Hanover, New Hampshire, United States of America; University of Chicago, United States of America

## Abstract

Molybdenum (Mo) is an essential micronutrient for plants, serving as a cofactor for enzymes involved in nitrate assimilation, sulfite detoxification, abscisic acid biosynthesis, and purine degradation. Here we show that natural variation in shoot Mo content across 92 *Arabidopsis thaliana* accessions is controlled by variation in a mitochondrially localized transporter (Molybdenum Transporter 1 - *MOT1*) that belongs to the sulfate transporter superfamily. A deletion in the *MOT1* promoter is strongly associated with low shoot Mo, occurring in seven of the accessions with the lowest shoot content of Mo. Consistent with the low Mo phenotype, *MOT1* expression in low Mo accessions is reduced. Reciprocal grafting experiments demonstrate that the roots of Ler-0 are responsible for the low Mo accumulation in shoot, and GUS localization demonstrates that *MOT1* is expressed strongly in the roots. MOT1 contains an N-terminal mitochondrial targeting sequence and expression of *MOT1* tagged with GFP in protoplasts and transgenic plants, establishing the mitochondrial localization of this protein. Furthermore, expression of *MOT1* specifically enhances Mo accumulation in yeast by 5-fold, consistent with MOT1 functioning as a molybdate transporter. This work provides the first molecular insight into the processes that regulate Mo accumulation in plants and shows that novel loci can be detected by association mapping.

## Author Summary

Plants must acquire all the mineral nutrients they require for survival from the complex chemical and biological environment of the soil. A better understanding of the way plants do this would not only allow improvements in sustainable agricultural productivity, but could also improve human health through enhancement of the nutritional quality of foods. One such essential mineral nutrient required by plants is molybdenum (Mo), which is needed as a cofactor in several critical biochemical reactions, including the utilization of nitrogen from the soil. By searching through numerous natural populations of *Arabidopsis thaliana* (Arabidopsis), we were able to identify a DNA deletion that drives the natural variation in Mo accumulation observed in these populations. This deletion reduces expression of a gene (*MOT1*) that the authors establish to encode a mitochondrially localized molybdenum transporter. Loss of expression of *MOT1* in the roots of Arabidopsis causes a significant reduction in whole plant Mo accumulation, though the mechanism by which this Mo transporter regulates whole plant Mo from the mitochondria remains to be established.

## Introduction

Plants have developed complex biochemical and regulatory pathways to acquire mineral nutrients from the soil environment and distribute them to appropriate tissues. Natural populations of *Arabidopsis thaliana* (Arabidopsis) provide an excellent system to study how plants have adapted their mineral nutrient and trace element uptake pathways to thrive under different environmental conditions. Molybdenum (Mo) is an important micronutrient for plants, being incorporated into molybdopterin, an essential cofactor for enzymes involved in nitrate assimilation, sulfite detoxification, abscisic acid biosynthesis and purine degradation [1]. Molybdenum in either deficiency or excess has been demonstrated to inhibit plant growth and agricultural productivity [2]. The genes comprising the biochemical pathway that synthesizes the molybdopterin cofactor have been identified in plants, animals and microbes, but to date, a Mo transporter in plants has not been found [2]. The first committed step in molybdopterin biosynthesis occurs in the mitochondria [3], confirming the predicted sub-cellular localization of the enzymes [1]. The remaining 3 steps are thought to occur in the cytoplasm [3]. While a substantial amount is known about the biochemistry, enzymology and underlying genetics of molybdopterin biosynthesis, very little is known about the mechanisms for Mo uptake, distribution and accumulation in plants. In this study, natural variation in whole plant Mo accumulation has been coupled with genomics techniques and genetics to identify a mitochondrial Mo transporter (*MOT1*) that regulates whole plant Mo content in Arabidopsis. Alleles of this gene are demonstrated to be responsible for low Mo accumulation across a diversity collection of 92 Arabidopsis accessions. All soil grown plant ionomic data from this study is freely available at www.purdue.edu/dp/ionomics [4].

## Results

**Figure 1:**
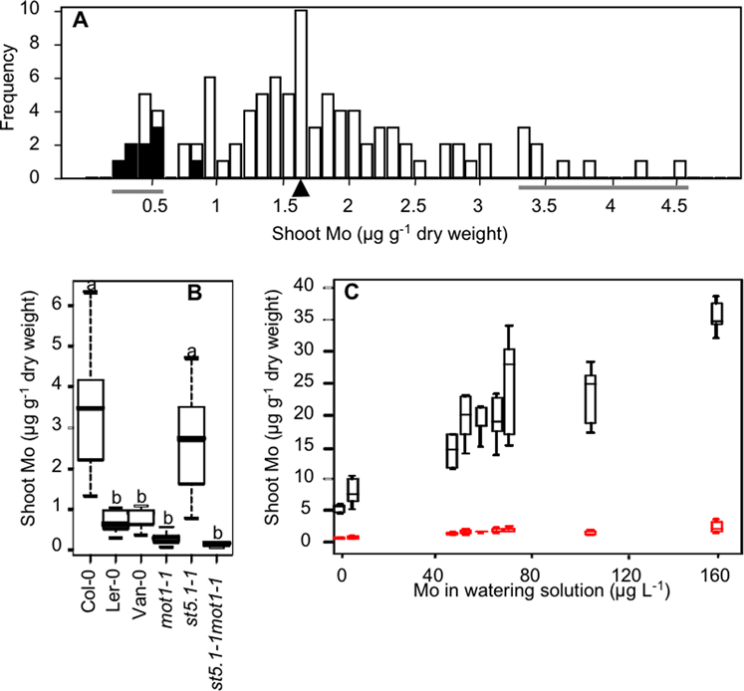
Genetic and physiological analysis of mo in arabidopsis. A: Shoot Mo content across 98 accessions. Histogram of shoot Mo content in 98 Arabidopsis accessions which include 94 from Nordborg et al. (2005). Black bars indicate the distribution of lines having the *mot1* ^Ler-0^ deletion. Grey lines denote the low and high accessions, as detailed in the text. The black arrow denotes Col-0 Mo content. Shoot Mo concentrations are normalized so that the average of the Col-0 and Cvi-0 means included in each growth tray are equivalent across all trays. Plants were grown in soil for 5 weeks. Data represents median values (average n = 11.6) for each accession. B: Mo accumulation of Col-0, Ler-0, Van-0, *mot1-1*, *st5.1-1* and the *mot1-1st5.1-1* double mutant. Data is shown as a five number summary (the minimum, 1^st^ quartile, median, 3^rd ^quartile and maximum) for each line, and is summarized from an average of 10 replicate plants for each line. Lower case letters denote groups that are not significantly different from each other at P<0.01 with the Holm correction. Plants were grown in soil for 5 weeks. C: Shoot Mo accumulation in Arabidopsis in response to increasing levels of Mo in the nutrient solution. Mo accumulation in Col-0 (Black) and Ler-0 (Red) after 5 weeks of growth in soil at varying concentrations of Mo in the watering solution. Data is shown as a five number summary for each line, and is summarized from 6 replicate plants for each treatment.

Shoot tissue from a geographically diverse panel of 98 Arabidopsis accessions (93 from [5] plus Bu-15, Col-4, Kas-1, Ler-0, Ler-2) was screened in an effort to identify accessions which have altered Mo content in shoot tissue (Figure 1A). Based on this survey it was determined that the shoot content of Mo in Arabidopsis is under genetic control (broad sense heritability H^2^ = 0.48), with an approximately 20-fold difference between the highest and lowest Mo levels observed (Figure 1A) across the diverse set of Arabidopsis accession tested. Shoot Mo content was found to be not normally distributed around the population mean (p<0.001), with 12 accessions (Ler-0, Ler-1, Ler-2, Van-0, Zdr-6, Kondara, KZ-9, Shadahara, Sorbo, Ll-0, Ts-5, Ws-0) accumulating less than 0.7 µg g^−1^ Mo, and 9 accessions (Ull2-5, Bur-0,Cvi-0, Nfa-10, Tamm-2, Tamm-27, Fab-2, Var2-1, Spr1-6) accumulating more than 3.1 µg g^−1^ Mo (grey bars in Figure 1A). We had previously identified Ler as a low Mo accumulating accession of Arabidopsis [6] and all Ler lines tested (which are likely genetically identical), were low in Mo. Shoot tissue of Ler-0 contains 70–80% less Mo compared to Col-0 (Figure 1B), and this difference from Col-0 is observed for Ler-0 when plants were grown with a broad range of Mo concentrations in the nutrient solution (Figure 1C). We selected this accession for further study due to the extensive genetic resources available for this accession, including the availability of genome sequence data and recombinant inbred lines (RIL), along with high density genotype data [7],[8].

**Figure 2:**
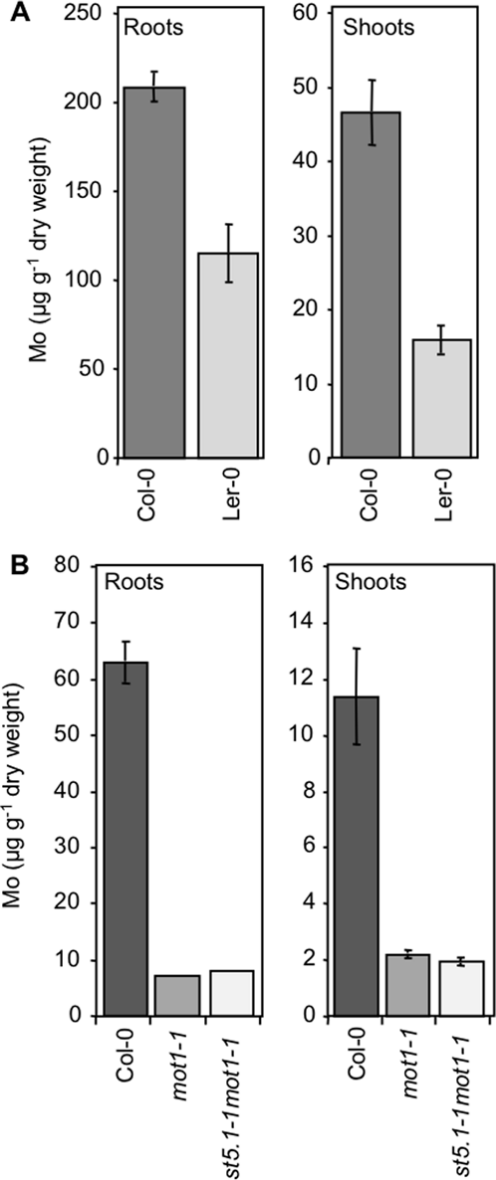
Mo content in the roots and shoots of plants grown hydroponically. Mo content in Col-0 and Ler-0 after 4 weeks growth (A), and Col-0, *mot1-1* and the *mot1-1st5.1-1* double mutant after 3-weeks growth (B). Presented data are the means of at least three biological replicates, and error bars represent±SE.

**Figure 3:**
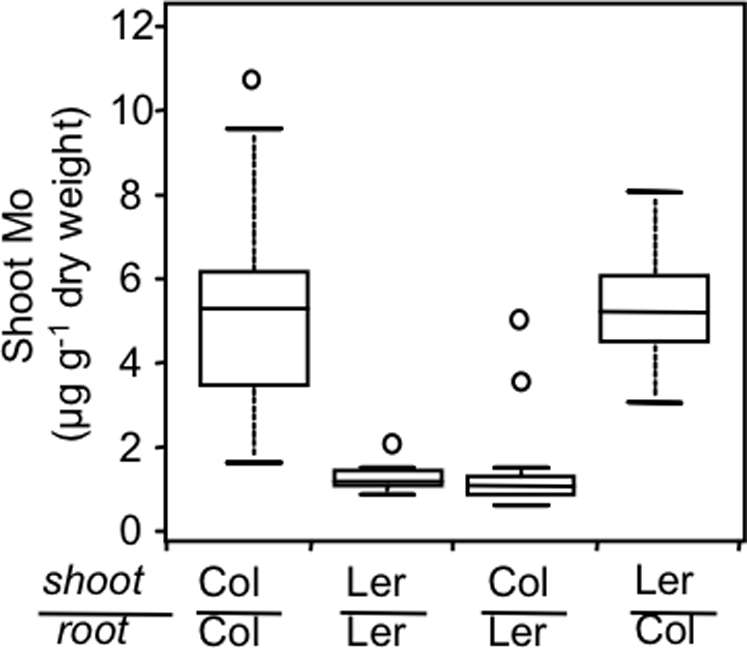
Shoot mo accumulation in Ler-0 is driven by the roots. Mo contents in Arabidopsis plants grafted at 5 days after germination and transferred to soil for growth were determined in self grafted plants of Col-0 (n = 32) and Ler-0 (n = 11), Col-0 shoot grafted onto Ler-0 root (n = 22), and Ler-0 shoot grafted onto Col-0 root (n = 15). Data is shown as a five number summary (the minimum, 1^st^ quartile, median, 3^rd^ quartile and maximum) for each line with outliers denoted by small circles.

The analysis of Mo in shoot and root tissue of hydroponically grown Ler-0 and Col-0 plants confirmed the low shoot Mo content of Ler-0 (Figure 2A). Furthermore, Ler-0 also has a reduced Mo content in roots when compared to Col-0, suggesting that the low shoot Mo content is not due to enhanced accumulation of Mo in the roots, but rather to reduced Mo uptake by the roots. Grafting experiments were performed to determine if low shoot Mo in Ler-0 is determined by the root or shoot. Shoots and roots from 5-day old seedlings of Ler-0 and Col-0 were reciprocally grafted, and grafted plants grown for 4 weeks in soil with short days. Plants with Ler-0 roots had significantly lower (p<0.01) shoot Mo contents than plants with Col-0 roots, whether the shoots were Ler-0 or Col-0. The genotype of the shoot stock had no significant effect on Mo content of the shoot (Figure 3). From this it was concluded that the low shoot Mo content of Ler-0 is driven solely by the roots.

### Mapping the Loci Responsible for the Low Mo Content of the Landsberg *Erecta* Arabidopsis Accession

**Figure 4:**
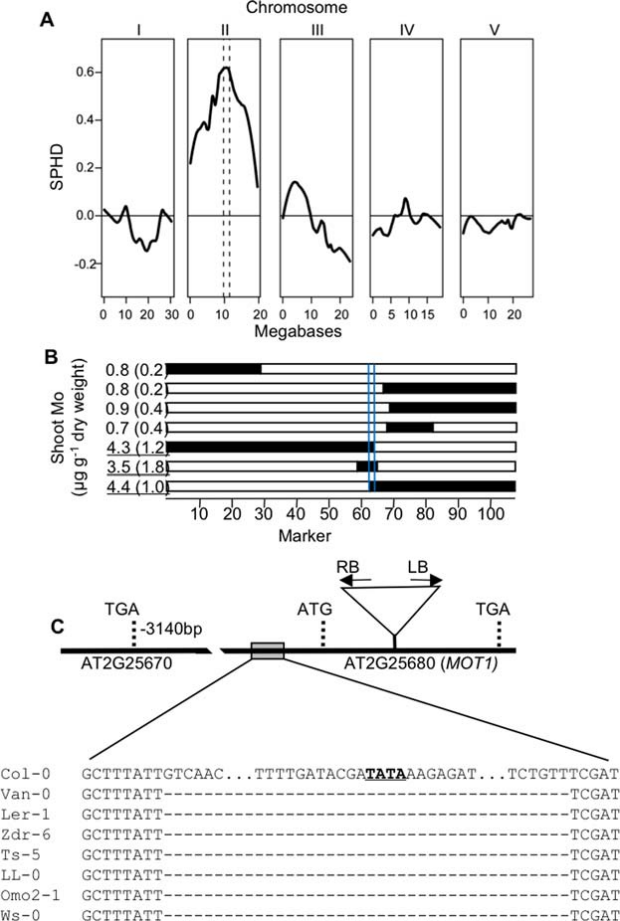
The locus determining low shoot Mo content in Ler-0 Maps to the *MOT1* Gene. A: Bulk Segregant analysis of the low shoot Mo content in an F2 population from a Col-0×Ler-0 cross.Data are presented as a scaled pool hybridization difference (SPHD), representing the difference between the hybridization of the two pools at the SFPs, scaled so that a pure Col-0 pool would be at −1 and a pure Ler-0 pool would be at 1. The pools were prepared from F2 plants with a low Mo content (n = 40) and F2 plants with a high Mo content (n = 40). SFPs were scored after hybridization of genomic DNA prepared from these pools to Affymetrix ATH1 DNA microarrays. Dotted lines denote likely location of the causal loci. B: Shoot Mo content and genotype on chromosome II of selected Col-0×Ler-0 RILs. The genotype of each of the 106 markers determined by Singer et al. (2006) for chromosome II are shown; Col-0 alleles are denoted in black, Ler-0 in white. The average shoot Mo content (n = 12) for each line is shown, and, those with Col-0 shoot levels of Mo are underlined. Blue lines indicate the narrowed mapping interval on chromosome II (10.771 to 11.056 Mb). C: Structure of the MOT1 gene. The DNA sequence of *MOT1* showing location of the T-DNA insert in *mot1-1*, and the 50 base pair deletion in the 5′ UTR in the seven accessions with low shoot Mo content aligned with the Col-0 DNA sequence, with the TATA box underlined.

In a Ler-0×Col-0 cross 51 of the 200 F_2_ progeny analyzed were found to have low shoot Mo contents similar to the Ler-0 parent. This ratio is consistent with the hypothesis that the low Mo phenotype in Ler-0 is controlled by a single locus, or several closely linked loci (p<0.00001 by Shapiro test for normality). Similar segregation patterns were observed in an F2 population derived from a Ler-0 cross to an ionomics mutant (*14501*) in the Col-0 background. To obtain a rough map position, a bulk segregant analysis (BSA) experiment [9] was performed with microarray detection of genetic markers [10],[11], using (14501[Col-0]×Ler-0) F_2_ plants. Plants with the lowest shoot Mo contents (n = 40) and plants with Mo shoot contents similar to Col-0 (n = 40) were pooled separately, and genomic DNA from each pool hybridized to the Affymetrix Arabidopsis ATH1 DNA microarray. Using the oligonucleotide probes on the DNA microarray which show differential hybridization between Ler-0 and Col-0 as genetic markers (Single Feature Polymorphisms or SFP), the locus responsible for the low shoot Mo content in Ler-0 was mapped to an area centered at 11 Mb on chromosome 2 (Figure 4A).

**Figure 5:**
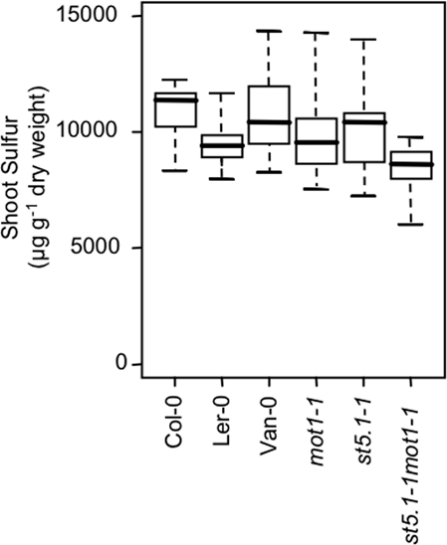
Sulfur accumulation in shoots of Col-0, Ler-0, Van-0, *mot1-1*, *st5.1-1* and the *mot1-1st5.1-1* double mutant plants. Data is shown as a five number summary (the minimum, 1^st^ quartile, median, 3^rd^ quartile and maximum) for each line, and is summarized from an average of 10 replicate plants for each line. No significant differences were observed. Plants were grown in soil for 5 weeks. The data is from the same plants as in Figure 1B.

**Figure 6:**
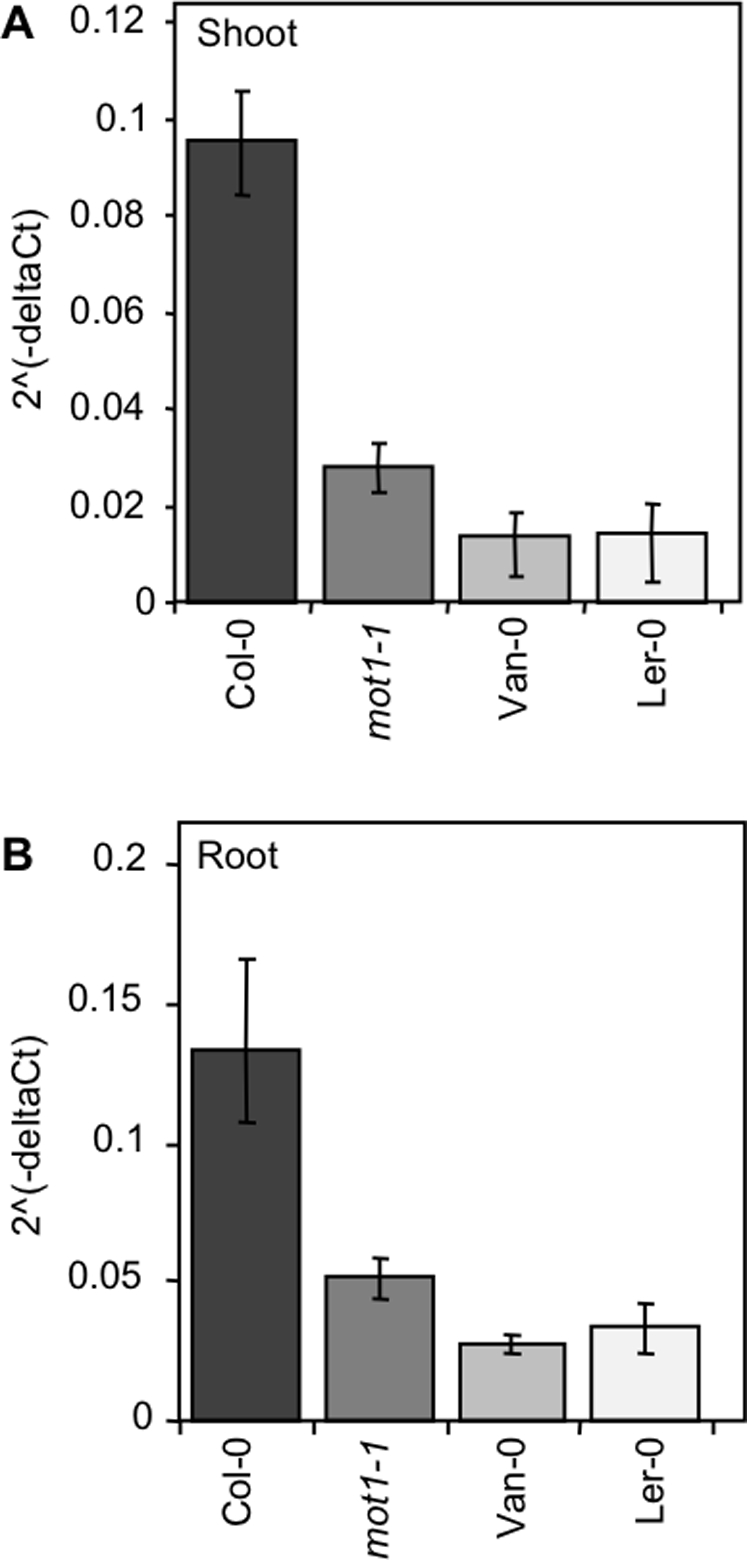
Expression of *MOT1* in Col-0, Ler-0, Van-0 and *mot1-1.* Steady state MOT1 expression level was compared in shoots (A) and roots (B) of Col-0, Ler-0, Van-0, and *mot1-1* using quantitative real time PCR (qRT-PCR). RNA was isolated from shoot and root of plants grown in soil for 5 weeks under short day conditions For normalization across samples expression of the *Actin 1* gene was used and the relative expression of *MOT1* calculated using the 2(−ΔCT) method. Presented data are the means of at least three biological replicates, (2ˆ(ΔCT)) each analyzed 4 times by qRT-PCR. Error bars represent±SD.

To further narrow down the map position of the locus controlling low shoot Mo content in Ler-0, the Col-4×Ler-0 RIL population [12] was used to identify seven recombinants in the mapping region we had previously determined from the BSA. DNA microarray-based genotyping of this Col-4×Ler-0 RIL set was used to further refine the break points between Col-4 and Ler-0 genotypes [7],[8]. The shoot Mo contents of these seven RILs showed a clear segregation with the genetic markers for Col-4 and Ler-0 within our mapping region, allowing us to classify these lines as having either the Col-0 or the Ler-0 allele for the low Mo locus (Figure 4B). The precise breakpoints identified by Singer et al. [8] allowed the mapping interval to be narrowed to 346 kb on chromosome 2 (10.771 to 11.056 Mb), an interval containing 81 genes. Candidate genes from this interval were selected based on annotation and expression differences between Col-0 and Ler-0 (T. Singer & S. Briggs, personal communication). T-DNA insertional alleles for these candidate genes were obtained, and the plants scored for low shoot Mo content compared to wild type Col-0. A null mutant (*mot1-1*) with an insertion disrupting the coding region of the gene At2g25680 (Salk_118311, Figure 4D), was observed to phenocopy the Ler-0 low shoot Mo content when grown in either soil (Figure 1B), or shoots and roots when grown hydroponically (Figure 2). Differences in the absolute concentrations of Mo between Col-0 in Figure 2A and 2B are related to differences in growth conditions between these two experiments. The At2g25680 gene was originally predicted to be a putative sulfate transporter, and named *AtSULT5.2* [13]. We have renamed this gene *MOT1* (molybdenum transporter 1) based on its phenotype of low shoot Mo content. Furthermore, we detected no change in the shoot content of S in *mot1-1* (Figure 5) or in the content of Li, B, Na, Mg, P, K, Ca, Mn, Fe, Co, Ni, Cu, Zn, As, Se, and Cd, providing additional support for the reannotation of *AtSULT5.2* as *MOT1*. Quantitative real-time RT-PCR of the *MOT1* transcript in *mot1-1* and Ler-0 revealed that both these low shoot Mo lines had significantly reduced expression levels of *MOT1* in both root and shoot tissue, compared to Col-0 (Figure 6). Van-0, a second low shoot Mo accession (Figure 1B) was also confirmed by qRT-PCR to have low expression of *MOT1* (Figure 6). Significantly, we observed no differences in shoot S content between Col-0 and either Ler-0 or Van-0 (Figure 5).

**Figure 7:**
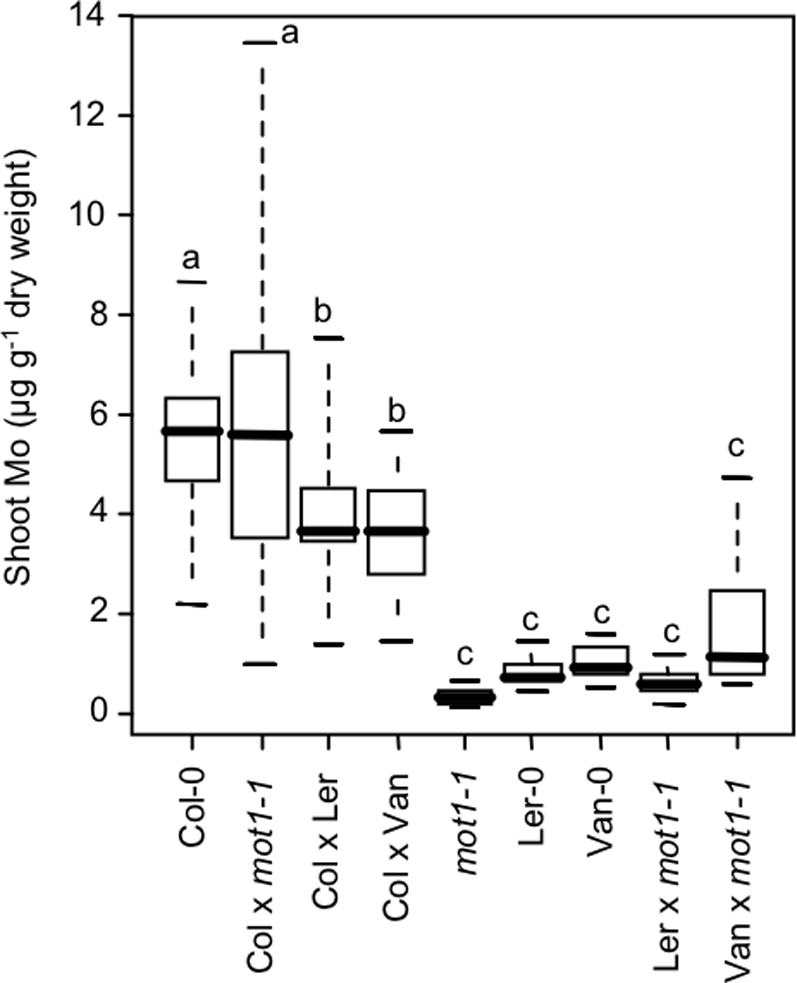
Complementation Studies Indicate MOT1 Natural Variant Is Responsible for Reduced Shoot Mo Content in Ler-0 and Van-0. Shoot Mo content of Col-0, Ler-0, Van-0, *mot1-1*, and F1 plants from crosses between Col-0×Ler-0, Col-0×Van-0, Ler-0×*mot1-1*, Van-0×*mot1-1*and Col-0×*mot1-*1 in 5-week old soil grown plants are shown. Data is represented as a five number summary (the minimum, 1^st^ quartile, median, 3^rd^ quartile and maximum) for each line, and is summarized from an average of 21 replicate plants for each line. Lower case letters denote groups that are not significantly different from each other at P<0.01 with the Holm correction.

To establish if *MOT1* is the locus responsible for the low shoot Mo content of Ler-0 and Van-0, we crossed *mot1-1* and Col-0 to both Ler-0 and Van-0 to test for complementation (Figure 7). F_1_ plants from the Ler-0×Col-0 and Van-0×Col-0 crosses contained significantly different levels of shoot Mo from either parent (p<0.005 for all pairwise t-tests), suggesting that the Col-0 allele of the low Mo locus could only partially complement the Ler-0 allele. F_1_ plants from the Ler-0×*mot1-1* and Van-0×*mot1-1* crosses were found to have significantly lower shoot Mo contents than similar plants from the Ler-0×Col-0 and Van-0×Col-0 crosses (p<0.0005). Such data establishes that *mot1-1* is deficient in complementing the low shoot Mo content of both Ler-0 and Van-0. This is strong evidence confirming that the Ler-0 and Van-0 alleles are allelic with the recessive loss of function allele of *MOT1.*

To determine the polymorphism in the *MOT1* ^Ler-0^ allele causal for low shoot Mo content, we sequenced *MOT1*, including 1 kb upstream and 200 bp downstream of the coding sequence in both Ler-0 and Van-0. We observed 18 polymorphisms in common between Ler-0 and Van-0, as well as several polymorphisms unique to each accession, when compared to the Col-0 reference sequence. These include 15 single nucleotide polymorphisms (SNPs) in the 1 kb upstream region, two SNPs that change two amino acids (I68T and V30L) in the coding region, and a 53 base pair (bp) deletion 27 nucleotides upstream from the translation start site (Text S1). The deletion includes the TATA box (Figure 4C). The altered expression of *MOT1* in both of the low shoot Mo accessions, Ler-0 and Van-0, suggested that this 5′ deletion may be the causal polymorphism driving low shoot Mo content in these accessions. To obtain further evidence that this deletion is the casual polymorphism driving low shoot Mo content, we performed an association analysis by sequencing the *MOT1* 5′ promoter region containing the 53 bp deletion in Ler-0 and Van-0 across 92 of the accessions originally screened for shoot Mo content (Text S2). Combining this information with the Nordborg [5] genotypes, we were able to scan for significant genetic associations with low shoot Mo content. The 53 bp deletion identified in Ler-0 and Van-0 was found in seven of the 92 accessions tested, and all accessions with the deletion had low shoot Mo content compared to the overall distribution of shoot Mo contents (Figures 4C and 1A). The distribution of p-values for genome-wide associations with shoot Mo contents were skewed towards significance, suggesting a relationship between Mo content and the underlying population structure (Figure S1), making the evaluation of individual loci difficult. However, when kinship and population structure were taken into account [14], the presence of the 53 bp deletion was found to be highly significantly associated with low shoot Mo content (p<0.0001) and accounted for ∼14% of the total variation in Mo accumulation. Once the *MOT1* ^Ler^ loci is accounted for, there were several other markers which showed significant associations with Mo accumulation, and these may represent additional loci of interest (Text S3).

**Figure 8:**
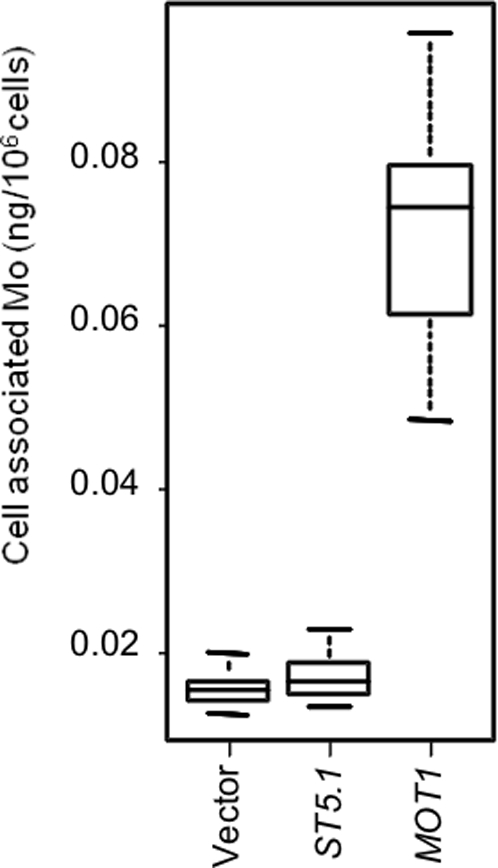
Cell Associated Mo in Yeast Expressing Arabidopsis *MOT1*. Cell associated Mo in yeast transformed with either p416 vector only or vector containing cDNAs encoding ST5.1 and MOT1 after growth for 24 hours. Data is represented as a five number summary (the minimum, 1^st^ quartile, median, 3^rd^ quartile and maximum) for each line, and is summarized from n = 32 replicate yeast cultures for each genotype.

To establish that MOT1 has the capacity to transport Mo, the Arabidopsis *MOT1* cDNA was expressed in yeast. Yeast expressing *MOT1* were observed to specifically accumulate five time more Mo than vector only controls (p<1E-14, Figure 8). Furthermore, the accumulation of no other element, including Na, Mg, P, S, K, Ca, Mn, Fe, Co, Ni, Cu, Zn, and Cd, was observed to be altered more than 20% in two independent experiments. This evidence is consistent with MOT1 being a specific molybdate transporter.

### Localization of *MOT1* Expression and Sub-Cellular Localization of the MOT1 Protein

**Figure 9:**
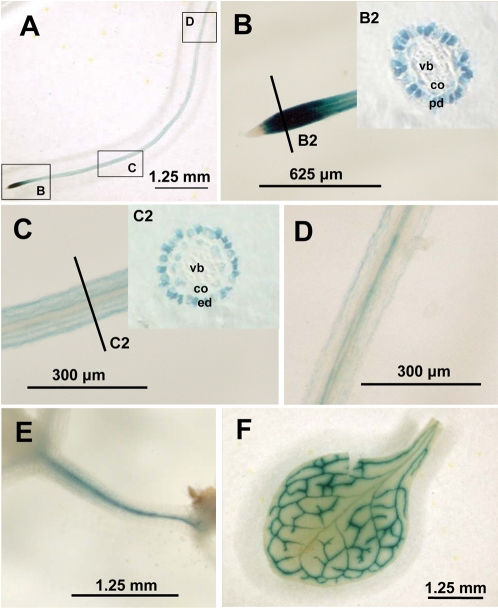
Tissue Localization of Expression of *MOT1* Using GUS Activity Visualized by Histochemical Staining. Pictures represent histochemical analysis of GUS activity in Arabidopsis plants stably transformed with a *MOT1*-promoter-GUS construct. A: Primary root shown from root tip to the beginning of the lateral root development. Boxes denote close-ups shown in B,C and D; B: Root tip and cross section shown in insert panel B2. C: Root elongation zone with a cross section shown in insert panel C2; D: Root shown from between the elongation zone and the start of the lateral root production zone; E: Hypocotyl; F: Fully expanded leaves. Pd – protoderm; co – cortex; vb vascular bundle; ep – epidermis.

To determine the tissue localization of *MOT1* expression, Col-0 was transformed with a 1.8 kb *MOT1* promoter-GUS construct. In all of the promoter-GUS lines examined, GUS staining was observed in the roots, hypocotyls and leaves (Figure 9). In roots, GUS staining was most pronounced just behind the growing root tip in the primary root (Figure 9A, B), and the lateral roots (data not shown). Cross sections show that the strong GUS staining behind the root tip was restricted mainly to the protodermal cells (Figure 9B2). At the beginning of the elongation zone, GUS staining was mainly restricted to the epidermis and cortex (Figure 9C2). Thereafter, GUS staining in the root also occurred in the vascular tissue (Figure 9D). In the hypocotyls the GUS staining was also mainly restricted to the vascular tissue (Figure 9E). In fully expanded leaves GUS staining was found in the vascular tissue. However, the main vein was less intensely stained than the lateral veins (Figure 9F). During flowering GUS activity was also visible in the vasculature of the stem leaves but not in the vasculature of the stem, flowers or developing siliques including seeds (data not shown).

**Figure 10:**
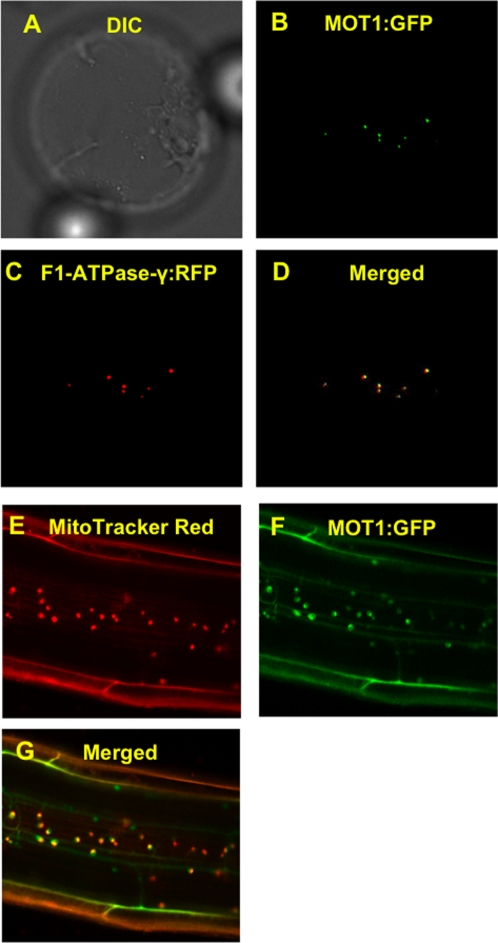
Subcellular localization of *MOT1*. *MOT1::GFP* was transiently expressed in Arabidopsis leaf protoplasts co transformed with F1-ATPase::RFP: (A) DIC (B) GFP filter (C) RFP filter and (D) merged image. Roots of Arabidopsis stably transformed with *MOT1::GFP* and stained with Mitotracker Red: (E) Red Filter (F) GFP Filter (G) Merged.

The sub-cellular localization of the MOT1 protein was determined by transiently expressing a MOT1 C-terminal GFP translational fusion construct in shoot derived Col-0 protoplasts (Figure 10A–10D), and by stable ectopic expression of a similar construct in Col-0 (Figure 10E–10G). The GFP signal was observed to co-localize with the mitochondrial marker F-ATPase–RFP in shoot derived protoplasts transiently expressing the construct, and with the Mitotracker Red dye in roots of the stably transformed lines. The experimentally determined mitochondrial localization for MOT1 is in good agreement with several sub-cellular prediction programs (summarized at the SUBA database, [15]), which predict a mitochondrial targeting sequence at the N-terminus of MOT1.

Phylogenetic analysis of the sulfate transporter family in Arabidopsis and rice [13],[16] reveal two Arabidopsis genes, *MOT1* and *ST5.1*, to have diverged significantly from the other sulfate transporter family members, and each Arabidopsis gene has a rice ortholog. Given the sequence similarity between *ST5.1* and *MOT1*, the shoot Mo content of a *st5.1-1* T-DNA insertion line (Salk_015044) was analyzed to determine if it also shows the low Mo phenotype observed in *mot1-1*. We were unable to detect any significant changes in the Mo content of shoot of *st5.1-1* when grown in soil and the roots and shoot Mo accumulation of a *mot1-1/st5.1-1* double mutant was not significantly different from the *mot1-1* mutant alone. (Figures 1B and 2B). The Mo content of yeast heterologously expressing the *ST5.1* cDNA was found not to be reproducibly significantly different from vector only controls in two independent experiments (Figure 8). Thus, we can find no evidence that ST5.1 is also a Mo transporter. A search of the PiiMS database [4]; www.purdue.edu/ionomics) found four T-DNA lines with insertions in sulfate transporters, representing the ST1, ST2 and ST3 subfamilies (At3g15990, At4g02700, At1g77990, At4g08620), none of which accumulated significantly different levels of Mo than Col-0.

## Discussion

Molybdenum is one of the 14 essential minerals required by plants. Despite its importance as a cofactor in processes ranging from nitrogen metabolism to hormone biosynthesis, we still know relatively little about the regulation, uptake and transport of this transition metal. Using an ionomics approach to identify genes that affect the elemental composition of plants, we identified *MOT1* as the causal gene driving reduced shoot Mo in various accessions of Arabidopsis. We have combined several lines of evidence to support this conclusion. First, a 54 bp deletion in the promoter of *MOT1* was found to be strongly associated (p<0.0001) with low shoot Mo content across 92 Arabidopsis accessions. Given the strong association between the presence of this deletion and low shoot Mo content, and the rate of linkage disequilibrium decay in Arabidopsis [17], if this deletion is not the causal polymorphism, it is within 20 kb of the causal polymorphism. Second, deficiency complementation with a T-DNA allele (SALK_118311) indicates that the *MOT1* allele found in Ler-0 and Van-0, accessions with a low content of shoot Mo, is responsible for this reduced Mo content. Third, *MOT1* shows reduced expression in both Ler-0 and Van-0 compared to Col-0, and the *mot1-1* null allele in the Col-0 background phenocopies the low shoot Mo observed in both Ler-0 and Van-0.

Given the fact that MOT1 belongs to the sulfate transporter superfamily, and can transport Mo when expressed in yeast, it is easy to imagine how reduced expression of a Mo transporter could lead to low shoot Mo content, either via reduction in uptake from the soil and/or translocation to the shoot. In Col-0, the functional *MOT1* allele is strongly expressed in the root differentiation zone and in mature vasculature tissue of both the roots and the shoots, suggesting a defect in either or both roles could be the possible explanation for the observed phenotype. Our grafting experiments clearly show that the Mo defect is associated with the roots. Surprisingly, the MOT1 protein is not localized to the plasma membrane of roots cells where it could function in uptake into cells, but rather MOT1 is localized to the mitochondria. Based on these observations, and the grafting data, it is hypothesized that MOT1 regulates whole plant Mo accumulation at the level of the mitochondria in the root. Interestingly, the first committed step in molybdopterin biosynthesis has recently been shown to occur in the mitochondria, and this is consistent with the mitochondria acting as a control point in regulating whole plant Mo content [3]. Given that characterized members of the sulfate transporter superfamily are SO_4_^−2^/H^+^ co-transporters, we speculate that MOT1 is transporting MoO_4_^−2^ from the acidic mitochondrial intermembrane space to either the cytoplasm or the matrix.

While we have localized MOT1 to the mitochondria, the question of how Mo enters the root cells remains. Based on its homology, *ST5.1-1* is a candidate for this role, however a ST5.1 C-terminal GFP fusion was found to localize to the vacuole (Buchner and Hawkesford, unpublished). Additionally, the *st5.1-1* T-DNA insertional mutant does not have altered shoot Mo content, and heterologous expression of *ST5.1* cDNA in yeast has no effect on Mo accumulation. *ST5.1* also appears not to interact with *MOT1* since the double mutant *st5.1mot1-1* showed the same reduction in Mo content as *mot1-1*.

Alternatively, the putative plasma membrane molybdate transporter could be from a family unrelated to the SUL gene family, as multiple gene families have been shown to transport Zn^2+^ and Ca^2+^, for example [18],[19]. Finally, it remains possible that MoO_4_^2−^ is transported across the plasma membrane through a promiscuous transporter(s) with broad ion specificity as shown for *E. coli* [20]. Both sulfate and phosphate starvation have been shown to increase Mo accumulation, which suggests that Mo may be transported across the plasma membrane by sulfate and phosphate transporters [21],[22].

Sequencing results show that the *MOT1* ^Ler-0^ allele has a frequency of approximately 7% (7/92) in the natural diversity collection of Arabidopsis obtained from populations collected from a broad geographical region. This frequency is higher than we would expect if the lowered Mo content strongly reduced fitness. For example, the overall reduction in Mo content might cause a reduction in molybdopterin, negatively impacting the activity of MoCo containing enzymes like nitrate reductase. However, we found no significant reduction in growth, N accumulation or nitrate reductase activity in *mot1-1* lines when grown with nitrate as the sole N source (data not shown). This suggests that MoCo levels are not limiting in *mot1-1*. Alternatively, loss of MOT1 function might increase the fitness of Arabidopsis by some as yet unknown process, and the association of Mo with population structure is consistent with Mo accumulation having a selective effect. However, an analysis of haplotype sharing [23] around this gene gave no indication of recent selection on the *MOT1* ^Ler-0^ allele (C. Toomajian, unpublished). Finally, it has been noted that large effect QTLs' might generally be too rare to be detected by association mapping [5],[17],[24]. The *MOT1* ^Ler-0 ^allele provides a clear counter-example, demonstrating that, given the dense marker maps that are now obtainable, association mapping will at least sometimes be a useful tool for finding such loci.

While this paper was in review, Tomatsu et al. [25] also reported that *MOT1* is the locus responsible for low Mo in Ler. However, they did not confirm this directly by complementation. Here we conclude that *MOT1* is the locus responsible for low Mo in Ler-0 and Van-0 based on genetic complementation, and further show that a deletion in the 5′ UTR of *MOT1* is strongly associated with low Mo in 92 different Arabidopsis accessions. The sequence for the *MOT1* ^Ler^ locus reported by Tomatsu et al. [25], derived from the Cereon resequencing project [7], differs from the results presented here by two additional amino acid altering SNPs. The *MOT1* ^Ler^ sequence presented here is based on resequencing after PCR amplification, and is consistent with that published by Clark et al. [26]. Furthermore, here we conclude that MOT1 is localized to the mitochondria. This is in contrast to Tomatsu et al. [25], who suggest that MOT1 is localized to the plasma membrane and the secretory and/or endocytic pathways. We attribute these inconsistencies to differences in constructs and localization systems used in the two studies. To determine the localization of MOT1 Tomatsu et al. [25] reported that they prepared a construct in which GFP was fused to the N-terminus of MOT1, blocking the predicted mitochondrial localization signal, and likely mislocalizing the GFP::MOT1 fusion protein in the tobacco BY-2 cells used for transient expression. Results reported here were obtained using a C-terminally fused MOT1::GFP construct that was expressed both transiently and stably in Arabidopsis and that clearly localized to the mitochondria.

In summary, natural variation has been used here to identify a mitochondrially localized Mo transporter that controls both root and shoot Mo content in Arabidopsis. This discovery demonstrates that natural accessions of Arabidopsis are a rich source of interesting alleles, useful for the functional characterization of genes of unknown function. Furthermore, the identification of MOT1 as a regulator of total plant Mo accumulation provides molecular insight into plant Mo homoeostasis.

Note added in proof: While this paper was in review, Tejada-Jimenez et al. [27] published the identification and characterization of the MOT1 ortholog in Chlamydomonas reinhardtii.

## Materials and Methods

### Plant Materials

All accessions were obtained from the ABRC or Lehle seeds. The insertion of the T-DNA into the *MOT1* coding region in *mot1-1* and into the *ST5.1* coding region in *st5.1-1* were verified by sequencing, and the mutant was confirmed to be null for *MOT1* expression by RT-PCR (Figure S2). All T-DNA lines analyzed were homozygous for the T-DNA insertion.

### General Plant Growth Conditions

Plants used for elemental profiling by ICP-MS analysis were grown in a controlled environment, 8 h light:16 h dark (90 µmol·m−2·s−1 light intensity) and 19 to 22°C [6]. Briefly, seeds were sown onto moist soil (Sunshine Mix LB2; Carl Brehob & Son, Indianapolis, Indiana, United States) with various elements added at subtoxic concentrations [As, Cd, Co, Li, Ni, Pb, and Se [6]] and stratified at 4°C for 3 d. Plants were bottom-watered twice per week with 0.25× Hoagland solution in which iron was replaced with 10 µM Fe-HBED [N,N′-di(2-hydroxybenzyl)ethylenediamine-N,N′-diacetic acid monohydrochloride hydrate; Strem Chemicals, Inc., http://www.strem.com). For elemental analysis after 5-weeks, plants were nondestructively sampled by removing one or two leaves. The plant material was rinsed with 18 MΩ water and placed into Pyrex digestion tubes. To alter the concentration of Mo in the watering solution, 0.25× Hoagland solution was made without any MoO_4_^2−^ which was then added back in varying concentrations from a solution of dissolved MoO_3_.

### Hydroponic Growth of Arabidopsis

At Purdue University seeds of Col-0 and Ler-0 were germinated in the dark at 4°C for 2 days on 0.5× Murashige and Skoog media with 0.5× MS Vitamins (Caisson Laboratories, Inc.), 3 mg/L Benomyl [methyl 1-(butylcarbamoyl)-2-benzimidazolecarbamate; Sigma, http://www.sigmaaldrich.com), and 10 µM Fe-HBED solidified with 1.5% agar in 1.5 ml eppendorf microcentrifuge tubes before being transferred into growth conditions described above. For the first 5 days after germinationthe tubes containing the seedlings were kept covered to maintain high humidity. The bottom of each tube was then removed and the tube inserted into foam floats placed in tubs containing ∼4 L of 0.25× Hoaglands solution with 10 µM Fe-HBED. The solution was changed weekly until harvest at 4 weeks from planting. Leaves were harvested as described above. The roots were rinsed twice in distilled water and a third time in double distilled water before being put in Pyrex digestion tubes. At Rothamsted seeds of Col-0, *mot1-1* and *st5.1mot1-1* were germinated on 0.5% agarose in 0.5 ml tubes (with excised lower portion) in racks placed on boxes (with transparent lids) filled with 700 ml nutrient solution: 1.0 mM KNO_3_, 0.5 mM Ca(NO_3_)_2_, 1.0 mM KH_2_PO_4_, 1.0 mM MgSO_4_, 100 µM FeEDTA, 30 µM H_3_BO_3_, 5 µM MnCl_2_, 1 µM ZnCl, 1 µM CuCl and 0.1 µM Na_2_MoO_4_. To synchronize germination, the boxes were incubated at 4°C over night and then transferred to a controlled growth room under light/dark cycle 16/8h at 20°C. The nutrition solution was exchanged 2 times per week. After 3 weeks, shoot and root materials were harvested. Roots were washed twice by dipping in deionised water and dried on paper towels before freezing in liquid nitrogen.

### Tissue Elemental Analysis

At Purdue University tissue samples were dried at 92°C for 20 h in Pyrex tubes (16×100 mm) to yield approximately 2–4 mg of tissue for elemental analysis. After cooling, seven of approximately 100 samples from each sample set were weighed. All samples were digested with 0.7 ml of concentrated nitric acid (OmniTrace; VWR Scientific Products; http://www.vwr.com), and diluted to 6.0 ml with 18 MΩ water. Elemental analysis was performed with an ICP-MS (Elan DRCe; PerkinElmer, http://www.perkinelmer.com) for Li, B, Na, Mg, P, S,K, Ca, Mn, Fe, Co, Ni, Cu, Zn, As, Se, Mo, and Cd. All samples were normalized to calculated weights, as determined with an iterative algorithm using the best-measured elements, the weights of the seven weighed samples, and the solution concentrations, implemented in the PiiMS database [4]. Alternatively, for the data shown in Figure 2B, samples were analyzed at Rothamsted. Frozen plant material was homogenized with a mortar and pestle in liquid nitrogen. After transfer into 2 ml tubes, the plant material was freeze-dried. Samples were acid digested (83% HNO_3_ & 13% (70%) HClO_4_) and analyzed by ICP-MS (Agilent ICP-MS 7500ce, Agilent Technologies, Santa Clara, CA, US) [28].

### Heritability and Association Analysis

Broad sense heritability was calculated using a general ANOVA to account for line and growth tray variation. A linear mixed model adjusting population structure confounding effects as in [14],[29] was used to test the marker-trait associations. A brief summary of the model is given below,
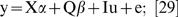
where y is the vector of phenotype, α is the vector of fixed allele effects, β is the vector of subpopulation effects, u is the vector of random effects reflecting the genome-wide relatedness, and X, Q, Z are known incidence matrices relating the observations to fixed and random effects, respectively.

The variance of phenotype was modeled as
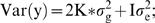
Thus, the phenotypic variance can be partitioned into two parts: σ^2^_g_, the genetic variance attributable to genome-wide effects, and σ^2^_e_, the residual variance.

The Q and K* matrix was the same as in [14], with Q being the population assignments by Structure [30] and K* being the kinship coefficient matrix, estimated as the proportion of shared haplotypes between individual pairs.

### DNA Microarray-Based BSA

DNA microarray-based BSA was realized as previously described [11],[31]. Briefly, SFPs were identified between Col-0 and Ler-0 by hybridizing labeled genomics DNA from each one of the accessions to Affymetrix ATH1 microarrays and comparing them to Col-0 hybridizations downloaded from http://www.naturalvariation.org/xam. Two genomic DNA pools from an F2 population of a cross between Ler-0 and the 14501 mutant in the Col-0 background were created and hybridized to separate DNA microarrays. Each one of the pools contained plants with either shoot Mo contents similar to Col-0 (“control” pool) or low shoot Mo contents similar to Ler (“Low Mo” pool). At loci unlinked to the low Mo phenotype, the pools should have equivalent amounts of each genotype, and the hybridization signal at each SFP should be intermediate between the two parent accessions, for an average difference between the two DNA microarrays of zero. At linked loci, the difference between the two DNA pools should be approximately two-thirds the difference between the parent accessions. By smoothing the signal across multiple SFPs, noise is reduced and the peak of the differences in hybridization signal will correspond to the chromosomal region of the loci controlling the low Mo trait. Raw hybridization data (.CEL files) for each probe on the ATH1 DNA microarrays used in these experiments have been submitted to the Gene Expression Omnibus (http://www.ncbi.nlm.nih.gov/geo) for public distribution (Reference #GSE10039).

### Grafting of Arabidopsis

Seedlings to be grafted as previously described by [10]. Plants were harvested for analysis of shoot Mo content 4 weeks after transfer to soil. Postharvest analysis of graft unions was performed under the stereoscope to identify any adventitious root formation from grafted individuals. Individuals with adventitious roots emerging at or above the graft union or without a clear graft union were eliminated from subsequent analyses.

### Quantitative Real-Time PCR

Plants were first analyzed by ICP-MS and further used to determine *MOT1* transcript levels as described previously by Rus et al., [10]. For *MOT1* (At2g25680) transcript quantification the following primers were used: forward primer 5′-GGT GGG TGT GTG GCA CTG T-3′ and reverse primer 5′-AGC ACA CCA ACC GGA AAC TT-3′. Four reactions were done per biological sample and three independent replicate samples per genotype were used. to evaluate the transcript abundance of *MOT1*. Data was analyzed using the SDS software (Applied Biosystems version 1.0), following the method of [32]. Ct values were determined based on efficiency of amplification. The mean Ct values were normalized against the corresponding *ACTIN 1* gene (At2g37620) and Ct values calculated as (Ct *_AtMOT1_*- Ct *_Actin1_*). The expression of *MOT1* was calculated as the 2^−ΔCt^ method. The final standard error was estimated by evaluating the 2^−ΔCt^ term using 2^−ΔCt^ plus standard deviation and 2^−ΔCt^ minus the standard deviation [32].

### Cloning and Sequencing of ***MOT1*** Genomic DNA

Genomic DNA was isolated from 10-day-old Arabidopsis Col-0 seedlings using a DNeasy plant mini kit (Qiagen, Valencia, CA). MOT1 clones containing approximately 1 kb upstream to the ORF, and approximately 0.250 kb downstream to the ORF were sequenced. The following primers were used to amplify different PCR products spanning the selected genomic region . Forward primers were: FP1 5′-CGA GCA AAC TAG AAA AGA GAT CG-3′, FP2 5′-CAG GTG TTA GCT GTT TAA CTG-3′, FP3-5′-GCG ATT TCG TCT ACC GC-3′ and FP4 5′-GGT TTG GAG CAA TGC C-3′. The corresponding reverse primers were: RP1 5′-CAA AAA CCA AAG CGT TGA CA-3′, RP2 5′-GGA CAC CGT AAA CTG C-3′ and RP3 5′-GGG AAG ATG TAG GTG G-3′. Conditions used in all PCR reactions were: initial denaturation 94°C followed by 30 cycles of 94° C 30 sec, 50° C 40 sec, 72° C 1 min 30 sec and final extension at 72° C for 10 min. All PCR products were cloned using TOPO XL PCR cloning kit (Invitrogen Corporation, Carlsbad, CA), and sequenced using Big dye terminator v 3.0 method (Applied Biosystems Foster city, CA) with T7 and universal M13 primers.

### Cloning of MOT1 cDNA

The MOT1 cDNA was isolated by RT-PCR. Oligonucleotide primers MOT1-5′ (5′-ATG GAG TCT CAG TCT CAG AGA GGT CAA-3′) and MOT1-3′ (5′-TCA AGC ATG TTC ACC GGA TTG CGG GGG-3′) were designed to amplify the coding sequence. Total RNA was extracted from roots of 4-week-old Arabidopsis plants grown on solidified one-half Murashige and Skoog + B5 vitamin medium using RNeasy Plant Mini Kit (Qiagen, Hilden, Germany). Reverse transcription was carried out at 50°C in 20 ul solution containing 1 ug total RNA, 0.5 ug oligo (dT) primer, 10 nmol dNTPs and 200 units Superscript II reverse transcriptase (Gibco BRL, Rockville, MD, USA). PCR was carried out on the first-strand cDNA using *Ex* Taq DNA polymerase (Takara) following the manufacturer's protocols. After a standard PCR of 30 cycles, aliquots were run on an agarose gel. The fragment of putative size was cloned into a *pGEM-T Easy* vector (Promega) and identified by DNA sequencing. For yeast expression studies, the MOT1 cDNA insert from *pGEM-T Easy* vector (Promega) was amplified using PCR with the primers (5′-CGG ACT AGT CCA TGG AGT CTC AGT CTC AGA GAG GT-3′) and (5′-TCC GGA TCC TCA AGC GTA ATC AGG AAC ATC GTA AGG GTA AGC ATG TTC ACC GGA TTG CGG GGG-3′), with an HA tag encoded by the reverse promoter. The fragments were cloned into p416 GPD (-uracil (ura)) (ATCC nos. 87360). Two separate MOT1::GFP fusion protein constructs were cloned, one at Purdue (v1) and one at Rothamsted (v2). To construct the MOT1::GFP(v1) fusion protein, *MOT1* was amplified from cDNA using PCR with 5′-TGC TCT AGA GCA TGG AGT CTC AGT CTC AGA GAG GT-3′ (MOT1, 5′ primer) and 5′-TCC GGA TCC TTC CTC CTC CAG CAT GTT CAC CGG ATT GCG GGG G-3′ (MOT1, 5′ primer) primers. The PCR product was confirmed by sequencing and cloned into the XbaI and BamHI sites of the 326-SGFP plasmid (kindly provide by Inhwan Hwang, POSTECH, Korea) to created chimeric GFP fusion constructs under the control of the 35S promoter.

### MOT1-Promoter-GUS and 35S-Promoter- MOT1::GFP (v2) Fusion Constructs

The use of the two specific oligonucleotide primers 5′-GGA TCC GCA GTC GAG CTT ACC AAT TCT C-3′ and 5′-GGA TCC GAA ACA GAG CAA TAA GCG TAT CTC-3′ allowed isolation of a 1778 bp MOT1-promoter DNA fragment (−1801 to −21 from the translation start site) from Arabidopsis Col-0 genomic DNA by PCR. After subcloning in pGEM-Teasy (Promega) and sequencing, the BamHI restriction sites in the primer sequences were used for further cloning of the promoter DNA fragment into the BamHI site of the pBI101 vector (Clontech, Mountain View, USA) harboring the promoterless β-glucuronidase gene. For preparation of the MOT1::GFP(v2) construct the full ORF without the stop codon (TGA) was amplified from total cDNA by using the two specific oligonucleotide primers 5′-GGA TCC AAT GGA GTC TCA GTC TCA GAG AG-3′ and 5′-GGA TCC AGC ATG TTC ACC GGA TTG CGG GG-3′. The Bam HI restriction site replacing the stop codon allowed the cloning of the coding DNA in frame to the GFP gene in the binary vector pBIN19 [33], which contained the cauliflower mosaic virus 35S-promoter-MCS-GFP cassette. The binary plasmids were transformed into *Agrobacterium tumefaciens* GV3101 (pM P90) [34] by the freeze/thaw method. Arabidopsis Col-0 plants were transformed using the floral dip method (Clough and Bent, 1998). Transgenic plants were selected on solidified ½ MS media [35] containing 50 mg/l kanamycin sulphate. Kanamycin-resistant homozygous T3 progenies derived from 5 independent transgenic lines were used for analysis.

### Cloning ST5.1 cDNA

Total RNA and first strand cDNA was isolated from Col-0 seedlings as described for qRT-PCR. The first strand cDNA was used as a template to synthesize *ST 5.1* cDNA using the following primers: SpeI-FP 5′-CGA CTA GTA TGG CGG TCG CAA TAT CTG GGA GT-3′ BamHI-HA-3′RP 5′-CGG GAT CCT TAA GCG TAA TCC GGA ACA TCA TAC GGG TAG TTT GCG AGT ATC GGG TT3′. The reverse primer contained the HA tag. PCR conditions are as follows: initial denaturation 94°C followed by 30 cycles of 94° C 30 sec, 50° C 40 sec, 72° C 1 min 20 sec and final extension at 72° C for 10 min. PCR products were cloned and sequenced as before. Plasmid DNA containing *ST5.1 HA* were digested with SpeI and BamHI and the cDNA fragment cloned into p416 GPD (Glycerol 3-phosphate dehydrogenase promoter) using the same restriction enzymes. The ligated products were chemically transformed into ***E. coli*** DH5**α** and the positive clones were identified using a vector primer (FP-5′-AAT GGA GTG ATG CAA CCT-3′) and the reverse primer BamHI-HA-3′RP HA.

### Histochemical Staining of MOT1 Promoter-GUS Plants

Transgenic Arabidopsis Col-0 plants were germinated and grown vertically on 0.5× MS agar plates containing 1% sucrose. Plants were transferred to 5 cm Petri dishes containing 2–4 ml staining solution, containing 0.05% 5-bromo 4 chloro 3 indolyl β-D glucuronic acid in 0.1 M sodium phosphate, 10 mM EDTA, 0.5 mM potassium ferricyanide, 0.5 mM potassium ferrocyanide, 0.3% triton X-100 and 10% methanol (pH 7.5). Vacuum infiltration for 1 min was performed twice and the staining reaction allowed to proceed in the dark at 37°C until the blue indigo color appeared. After straining plant samples were rinsed twice in 70% ethanol for 30 min then in 100% ethanol until chlorophyll was removed. After staining and destaining samples were analysed by optical microscopy.

### Subcellular Localization of MOT1 in Arabidopsis

The MOT1::GFP(v1) construct and a mitochondria marker, F1-ATPase-γ-RFP [36], were co-transformed into purified Arabidopsis leaf protoplasts using polyethyleneglycol method [37]. The protoplasts were incubated for 20 hours at 22°C, and examined under an epi-fluorescence microscope, Nikon Eclipse 80i (Nikon USA, Melville, NY). Images of the mitochondria marker and the green fluorescence of MOT1::GFP were sequentially taken within 1 second in the same cell, because plant mitochondria actively moves along F-actins [38]. The filter sets used were 31303 for RFP and 31001 for GFP from Chroma Technol Corp (Rockingham, VT).

The plants containing stably transformed 35S-Promoter-MOT1::GFP (v2) fusion constructs described above were grown vertically on B5 plates under continuous light for two weeks. The seedlings were harvested, and incubated with 0.2 µM MitoTracker Red CM-H_2_X ROS (Invitrogen, Carlsbad, CA) for 10 min at room temperature, and examined using a confocal microscope, Nikon Eclipse 80i (Nikon USA, Melville, NY) using green HeNe laser line (543 nm) for the GFP and red HeNe laser line (633 nm) for MitoTracker.

### Functional Characterization of MOT1 in Yeast

The MOT1-HA and ST5.1-HA containing plasmids were transferred into the *Saccharomyces cerevisiae* yeast wild-type strain BY4742 (*MATα his3 leu2 lys2 ura3*) by the lithium acetate method [39]. The transformants were selected on SD minimal media containing 20 g l^−1^ glucose or glactose and required amino acids. Yeast transformants were first pre-grown at 30°C and 320 rpm in four different culture tubes per construct for one day in SD URA- minimal media. Each independent culture was used to inoculate 8 wells of a 2 mL square deep well 96-well plate (600 µL per well defined minimal growth medium inoculated with 20 µL of yeast culture). The plate was covered with a breathable seal and incubated at 30°C with shaking at 400 rpm for 24 hr. An AcroPrep® 96 PVDF (Polyvinylidene fluoride) filter membrane (0.45 µm, 350 µL) micro well plate (Pall Life Sciences) was wetted by partially filling with methanol and filtered using a vacuum block. Wells were then filled with deionized water and similarly filtered. Yeast culture samples, grown in the 2 ml deep well 96-well plates, were transferred to the 96-well filter plates (200 µL/well) and the yeast growth media removed by filtration. The same amount of culture samples was concurrently transferred into Clear View® micro plate (Whatman) for optical density measurement with a Dynex Opsys MR plate reader. Yeast cells on the membrane were washed four times, respectively, with 1 µM EDTA, pH 8, and deionized water. 96-well filter plates were dried at 88°C for 2 h. Concentrated nitric acid (100 µL/well) was added, the plate covered with polypropylene lid, and placed in a preheated heating block set at 88°C for 35 min to digest the yeast cells. The digested samples were diluted with a gallium internal standard (5 ppb final concentration) and filtered into a 2-mL square deep well 96-well plate containing Triton X-100 (0.025%, 300 µL). The final dilution volume was 1.6 mL, giving an acid and Triton X-100 concentration of about 6.5% and 0.005%, respectively. The filtered culture medium, as well as the original medium, was diluted 50 times but otherwise the matrix prepared in the same manner as the samples. The samples and the media were analyzed on a Perkin Elmer Elan DRC-e ICP-MS coupled with Elemental Scientific SC-2 autosampler and Apex Q nebulizer sample introduction system, and the following analytes quantified: Na, Mg, P, S, K, Ca, Mn, Fe, Co, Ni, Cu, Zn, Mo and Cd. Solution concentrations for all yeast sampleswere normalized to the measured optical densities of the corresponding yeast culture. The amount of yeast was converted from OD to number of cells using the conversion factor of 1ml of 1 OD culture containing 3×10^7^ cells.

### Accession Numbers

The Arabidopsis Biological resource Center (ABRC) accessions used in this paper were CS22660, CS20, CS1965, CS1968, CS1971, CS1986,CS1925, CS1939,CS1975.

## Supporting Information

Figure S1 The Cumulative Distribution of p-Values in Genome-Wide Scans for Shoot Mo Accumulation. The cumulative distribution function of (cdf) of p-values for Mo accumulation across the genome with (mixed model) and without (naïve) correcting for population structure.
(0.07 MB TIF)

Figure S2 RT-PCR Amplification of the Full Length *MOT1* cDNA in *mot1-1* and Full Length *ST5.1* cDNA in *st5.1-1*. Forward and reverse 20 bp primer were used that contained the ATG start codon and the TGA-stop codon.
(0.04 MB TIF)

Text S1 Alignment of MOT1 from Col-0, Ler-0 and Van-0, including sequence 1kb upstream and 250bp downstream of the open reading frame.
(0.05 MB DOC)

Text S2 Arabidopsis accessions used in the association mapping study.
(0.03 MB DOC)

Text S3 Significant genotype vs shoot Mo associations.
(0.02 MB DOC)
