# Supplementary figures and images for: Variation in Molybdenum Content Across Broadly Distributed Populations of Arabidopsis thaliana Is Controlled by a Mitochondrial Molybdenum Transporter (MOT1)
Source: PLoS Genet. 2008 Feb 29;4(2):e1000004. doi: 10.1371/journal.pgen.1000004 (PMC2265440; doi:10.1371/journal.pgen.1000004)

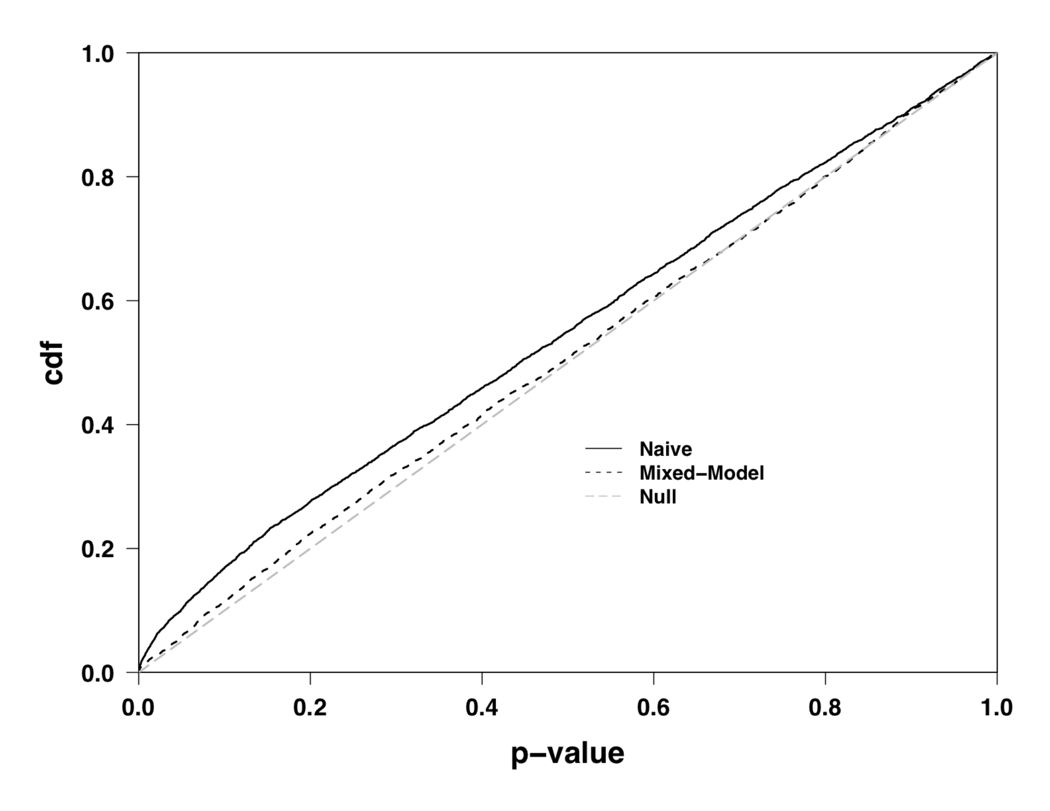

Supplement: Figure S1 — The Cumulative Distribution of p-Values in Genome-Wide Scans for Shoot Mo Accumulation. The cumulative distribution function of (cdf) of p-values for Mo accumulation across the genome with (mixed model) and without (naïve) correcting for population structure. (0.07 MB TIF) [file pgen.1000004.s001.tif]

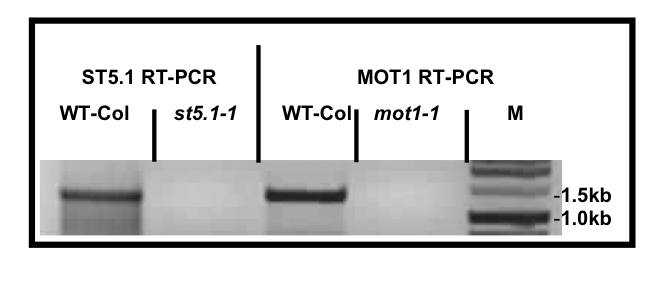

Supplement: Figure S2 — RT-PCR Amplification of the Full Length MOT1 cDNA in mot1-1 and Full Length ST5.1 cDNA in st5.1-1. Forward and reverse 20 bp primer were used that contained the ATG start codon and the TGA-stop codon. (0.04 MB TIF) [file pgen.1000004.s002.tif]
