# Supplementary material for: Variation in Molybdenum Content Across Broadly Distributed Populations of Arabidopsis thaliana Is Controlled by a Mitochondrial Molybdenum Transporter (MOT1)
Source: PLoS Genet. 2008 Feb 29;4(2):e1000004. doi: 10.1371/journal.pgen.1000004 (PMC2265440; doi:10.1371/journal.pgen.1000004)
Supplement: Text S1 — Alignment of MOT1 from Col-0, Ler-0 and Van-0, including sequence 1kb upstream and 250bp downstream of the open reading frame. (0.05 MB DOC) [file pgen.1000004.s003.doc]

Supplemental Figure 1: Forward and reverse primers are underlined. Forward and reverse primer sequences are from Col-0 (AT2G25680) sequence. The start and stop codons are highlighted in yellow.

1 50

Van-0 MOT1 (1) CGAGCAAACTAGAAAAGAGATCGACCATCATCTCTAGGATCAATCGATCT

Ler-0 MOT1 (1) CGAGCAAACTAGAAAAGAGATCGACCATCATCTCTAGGATCAATCGATCT

Col-0 MOT1 (1) CGAGCAAACTAGAAAAGAGATCGACCATCATCTCTAGGATCAATCGATCT

Consensus (1) CGAGCAAACTAGAAAAGAGATCGACCATCATCTCTAGGATCAATCGATCT

51 100

Van-0 MOT1 (51) CAACATCTATACTTGAGAATTTTAAAAATATTTCAACTTTGATGCTGATT

Ler-0 MOT1 (51) CAACATCTATACTTGAGAATTTTAAAAATATTTCAACTTTGATGCTGATT

Col-0 MOT1 (51) CAACATCTATACTTGAGAATTTTAAAAATATTTCAACTTTGATGCTGATT

Consensus (51) CAACATCTATACTTGAGAATTTTAAAAATATTTCAACTTTGATGCTGATT

101 150

Van-0 MOT1 (101) CATCTCATCTTTTTTAGAGGATTTTCACATTTATAAAAGGCTTATTGGAT

Ler-0 MOT1 (101) CATCTCATCTTTTTTAGAGGATTTTCACATTTATAAAAGGCTTATTGGAT

Col-0 MOT1 (101) CATCTCATCTTTTTTAGAGGATTTTCACATTTATAAAAGGCTTATTGGAT

Consensus (101) CATCTCATCTTTTTTAGAGGATTTTCACATTTATAAAAGGCTTATTGGAT

151 200

Van-0 MOT1 (151) GCCTAACTGATTCTATCCGAAATTCATTCGGTTCCTATAAAGTAAAAACG

Ler-0 MOT1 (151) GCCTAACTGATTCTATCCGAAATTCATTCGGTTCCTATAAAGTAAAAACG

Col-0 MOT1 (151) GCCTAACTGATTCTATCCGAAATTCATTCGGTTCCTATAAAGTAAAAACG

Consensus (151) GCCTAACTGATTCTATCCGAAATTCATTCGGTTCCTATAAAGTAAAAACG

201 250

Van-0 MOT1 (201) AATCTTAAACTCTCAACCCAAAATCTCAAAATTTGAATTACTCAATATAA

Ler-0 MOT1 (201) AATCTTAAACTCTCAACCCAAAATCTCAAAATTTGAATTACTCAATATAA

Col-0 MOT1 (201) GATCTTAAACTCTTAACCGGAAATCTCAAAATTTGAATTACTCAATATAA

Consensus (201) AATCTTAAACTCTCAACCCAAAATCTCAAAATTTGAATTACTCAATATAA

251 300

Van-0 MOT1 (251) AATAATTCGTGAAACCCGAATGTTCAAGACTACCGGAAACATCTGTGAAA

Ler-0 MOT1 (251) AATAATTCGTGAAACCCGAATGTTCAAGACTACCGGAAACATCTGTGAAA

Col-0 MOT1 (251) AATAATTCGTGAAACCCGAATGTTCAAGACTACCGGAAACATCTGTGAAA

Consensus (251) AATAATTCGTGAAACCCGAATGTTCAAGACTACCGGAAACATCTGTGAAA

301 350

Van-0 MOT1 (301) TATAAAGCTTTGTTTGTTAATATTACTTCCTCTGTTTCACCAAAGTATCA

Ler-0 MOT1 (301) TATAAAGCTTTGTTTGTTAATATTACTTCCTCTGTTTCACCAAAGTATCA

Col-0 MOT1 (301) TATAAAGCTTTATTTGTTAATATTACTTCCTCTGTTTCACCAAAGTATCA

Consensus (301) TATAAAGCTTTGTTTGTTAATATTACTTCCTCTGTTTCACCAAAGTATCA

351 400

Van-0 MOT1 (351) CAAAAATTAATAAAACTAAATTTCTACCATATGCTTAATGGGTTTTGTAT

Ler-0 MOT1 (351) CAAAAATTAATAAAACTAAATTTCTATCATATGCTTAATGGGTTTTGTAT

Col-0 MOT1 (351) CAAAAATTAATAAAACTAAATTTCTATTATATGCTTAATGGGTTTTGTGT

Consensus (351) CAAAAATTAATAAAACTAAATTTCTATCATATGCTTAATGGGTTTTGTAT

401 450

Van-0 MOT1 (401) AAAATAATAATTATTTTCTAACTAATAAAATGAAACAATAGACATAGGAA

Ler-0 MOT1 (401) AAAATAATAATTATTTTCTAACTAATAAAATGAAACAATAGACATAGGAA

Col-0 MOT1 (401) AAAATAATAATTATTTTCTAACTAATAAAATGAAACAATAGAGATAGGAA

Consensus (401) AAAATAATAATTATTTTCTAACTAATAAAATGAAACAATAGACATAGGAA

451 500

Van-0 MOT1 (451) ATGATACTTAATCTTACAAATTTAACTAATAGTACAAAGTACAAACATTA

Ler-0 MOT1 (451) ATGATACTTAATCTTACAAATTTAACTAATAGTACAAAGTACAAACATTA

Col-0 MOT1 (451) ATGATACTTAATCTTACAAATTTAACTAATAGTACAAAGTACAAACATTA

Consensus (451) ATGATACTTAATCTTACAAATTTAACTAATAGTACAAAGTACAAACATTA

501 550

Van-0 MOT1 (501) TTATTTTAAAACAAAAAAAAAA-CTTGAAAACAACTTTGTAAAACAGAAT

Ler-0 MOT1 (501) TTATTTTAAAACAAAAAAAAAA-CTTGAAAACAACTTTGTAAAACAGAAT

Col-0 MOT1 (501) TTATTTTAAAACAAAAAAAAAAACTTGAAAACAACTTTGTAAAACAGAAT

Consensus (501) TTATTTTAAAACAAAAAAAAAA CTTGAAAACAACTTTGTAAAACAGAAT

551 600

Van-0 MOT1 (550) AAATATTAAAATTAATTTATTTATTTACTTGATGGCGACTTTTTCATTGC

Ler-0 MOT1 (550) AAATATTAAAATTAATTTATTTATTTACTTGATGGCGACTTTTTCATTGC

Col-0 MOT1 (551) AAATATTAAAATTAATTTATTTATTTACTTGATGGCGACTTTTTCATTGC

Consensus (551) AAATATTAAAATTAATTTATTTATTTACTTGATGGCGACTTTTTCATTGC

601 650

Van-0 MOT1 (600) CGGTTCAAAACTCAAATAATAAATACATATCTCTTATTTGTATATAAGTG

Ler-0 MOT1 (600) CGGTTCAAAACTCAAATAATAAATACATAGCTCTTATTTGTATATAAGTG

Col-0 MOT1 (601) CGGTTCAAAACTCAAATAATAAATACACAGCTCTTATTTGTATATAAGTG

Consensus (601) CGGTTCAAAACTCAAATAATAAATACATAGCTCTTATTTGTATATAAGTG

651 700

Van-0 MOT1 (650) TTGACTTTTGATATATATAAATACCTTTCCATAAATGAATAATTTATGTT

Ler-0 MOT1 (650) TTGACTTTTGATATATATAAATACCTTTCCATAAATGAATAATTTATGTT

Col-0 MOT1 (651) TTGACTTTTGATTTATATAAATATCTTTCCATAAATGAATAATTTAAGTT

Consensus (651) TTGACTTTTGATATATATAAATACCTTTCCATAAATGAATAATTTATGTT

701 750

Van-0 MOT1 (700) GCTTAGATTTATATAAATAACCTACAAATTCAGGTGTTAGCTGTTTAACT

Ler-0 MOT1 (700) GCTTAGATTTATATAAATAACCTACAAATTCAGGTGTTAGCTGTTTAACT

Col-0 MOT1 (701) GCTTAGATTTATATAAATCATCTACAAATTCAGGTGTTAGCTGTTTAACT

Consensus (701) GCTTAGATTTATATAAATAACCTACAAATTCAGGTGTTAGCTGTTTAACT

751 800

Van-0 MOT1 (750) GAAGATATAATAAAAATATGTGCCAGTATTAAATTCGTACAAAAATTAGT

Ler-0 MOT1 (750) GAAGATATAATAAAAATATGTGCCAGTATTAAATTCGTACAAAAATTAGT

Col-0 MOT1 (751) GAAGATATAATAAAAATATGTGCCAGTATTAAATTCGTACAAAAATTAGT

Consensus (751) GAAGATATAATAAAAATATGTGCCAGTATTAAATTCGTACAAAAATTAGT

801 850

Van-0 MOT1 (800) TGACGATAAATAAATCCTAATTCGTACAACTAAGTACAAAAAATTAGTTG

Ler-0 MOT1 (800) TGACGATAAATAAATCCTAATTCGTACAACTAAGTACAAAAAATTAGTTG

Col-0 MOT1 (801) TGACGATAAATAAATCCTAATTCGTACAACTAAGTACAAAAAATTAGTTG

Consensus (801) TGACGATAAATAAATCCTAATTCGTACAACTAAGTACAAAAAATTAGTTG

851 900

Van-0 MOT1 (850) ACGATAAATAAATTAGTTGACGATAAAGGATAATTTTGATAAGTTTAAGG

Ler-0 MOT1 (850) ACGATAAATAAATTAGTTGACGATAAAGGATAATTTTGATAAGTTTAAGG

Col-0 MOT1 (851) ACGATAAATAAATTAGTTGACGATAAAGGATAATTTTGATAAGTTTAAGG

Consensus (851) ACGATAAATAAATTAGTTGACGATAAAGGATAATTTTGATAAGTTTAAGG

901 950

Van-0 MOT1 (900) CAAATTTTTTCTAACACACAATAAAGAATCAGATACTGTCGCCATCAAGG

Ler-0 MOT1 (900) CAAATTTTTTCTAACACACAATAAAGAATCAGATACTGTCGCCATCAAGG

Col-0 MOT1 (901) CAAATTTTTTCTAACACACAATAAAGAATCAGATACTGTCGCCATCAAGG

Consensus (901) CAAATTTTTTCTAACACACAATAAAGAATCAGATACTGTCGCCATCAAGG

951 1000

Van-0 MOT1 (950) TTTTGCTTTATT--------------------------------------

Ler-0 MOT1 (950) TTTTGCTTTATT--------------------------------------

Col-0 MOT1 (951) TTTTGCTTTATTGTCAACGCTTTGGTTTTTGATACGATATAAAGAGATAC

Consensus (951) TTTTGCTTTATT

1001 1050

Van-0 MOT1 (962) ---------------TCGATACAAACCACAAAACAGAAACAATGGAGTCT

Ler-0 MOT1 (962) ---------------TCGATACAAACCACAAAACAGAAACAATGGAGTCT

Col-0 MOT1 (1001) GCTTATTGCTCTGTTTCGATACAAACCACAAAACAGAAACAATGGAGTCT

Consensus (1001) TCGATACAAACCACAAAACAGAAACAATGGAGTCT

1051 1100

Van-0 MOT1 (997) CAGTCTCAGAGAGGTCAACACGAAACCCCGAAACGTTCTAGGTTCACCGG

Ler-0 MOT1 (997) CAGTCTCAGAGAGGTCAACACGAAACCCCGAAACGTTCTAGGTTCACCGG

Col-0 MOT1 (1051) CAGTCTCAGAGAGGTCAACACGAAACCCCGAAACGTTCTAGGTTCACCGG

Consensus (1051) CAGTCTCAGAGAGGTCAACACGAAACCCCGAAACGTTCTAGGTTCACCGG

1101 1150

Van-0 MOT1 (1047) AATGTTCCATAAACTGAAAACGAATCTTCTTTTCCGGTCGAAGCTAGCCG

Ler-0 MOT1 (1047) AATGTTCCATAAACTGAAAACGAATCTTCTTTTCCGGTCGAAGCTAGCCG

Col-0 MOT1 (1101) AATGTTCCATAAACTGAAAACGAATCTTGTTTTCCGGTCGAAGCTAGCCG

Consensus (1101) AATGTTCCATAAACTGAAAACGAATCTTCTTTTCCGGTCGAAGCTAGCCG

1151 1200

Van-0 MOT1 (1097) AAATAAACGGTGCAATGGGTGATCTTGGTACTTACATACCAATCGTCCTC

Ler-0 MOT1 (1097) AAATAAACGGTGCAATGGGTGATCTTGGTACTTACATACCAATCGTCCTC

Col-0 MOT1 (1151) AAATAAACGGTGCAATGGGTGATCTTGGTACTTACATACCAATCGTCCTC

Consensus (1151) AAATAAACGGTGCAATGGGTGATCTTGGTACTTACATACCAATCGTCCTC

1201 1250

Van-0 MOT1 (1147) GCTTTAACTCTAGCCAAGGATTTGGATTTAGGCACAACACTGACATCCAC

Ler-0 MOT1 (1147) GCTTTAACTCTAGCCAAGGATTTGGATTTAGGCACAACACTGACATTCAC

Col-0 MOT1 (1201) GCTTTAACTCTAGCCAAGGATTTGGATTTAGGCACAACACTGATATTCAC

Consensus (1201) GCTTTAACTCTAGCCAAGGATTTGGATTTAGGCACAACACTGACATTCAC

1251 1300

Van-0 MOT1 (1197) CGGCATATACAACGCGATAACCGGAGCAGTTTACGGTGTCCCCATGCCGG

Ler-0 MOT1 (1197) CGGCATATACAACGCGATAACCGGAGCAGTTTACGGTGTCCCCATGCCGG

Col-0 MOT1 (1251) CGGCATATACAACGCGATAACCGGAGCAGTTTACGGTGTCCCCATGCCGG

Consensus (1251) CGGCATATACAACGCGATAACCGGAGCAGTTTACGGTGTCCCCATGCCGG

1301 1350

Van-0 MOT1 (1247) TTCAACCGATGAAATCGATAGCAGCCGTGGCGATTTCGTCTACCGCGGAA

Ler-0 MOT1 (1247) TTCAACCGATGAAATCGATAGCAGCCGTGGCGATTTCGTCTACCGCGGAA

Col-0 MOT1 (1301) TTCAACCGATGAAATCGATAGCAGCCGTGGCGATTTCGTCTACCGCGGAA

Consensus (1301) TTCAACCGATGAAATCGATAGCAGCCGTGGCGATTTCGTCTACCGCGGAA

1351 1400

Van-0 MOT1 (1297) GATTTCGGTATACCGGAGATTATGGCTGCCGGAATATGTACCGGAGGGAT

Ler-0 MOT1 (1297) GATTTCGGTATACCGGAGATTATGGCTGCCGGAATATGTACCGGAGGGAT

Col-0 MOT1 (1351) GATTTCGGTATACCGGAGATTATGGCTGCCGGAATATGTACCGGAGGGAT

Consensus (1351) GATTTCGGTATACCGGAGATTATGGCTGCCGGAATATGTACCGGAGGGAT

1401 1450

Van-0 MOT1 (1347) CTTGTTCGTGTTGGGGATCTCTGGTTTGATGCAGCTTGTGTTCAATATAA

Ler-0 MOT1 (1347) CTTGTTCGTGTTGGGGATCTCTGGTTTGATGCAGCTTGTGTTCAATATAA

Col-0 MOT1 (1401) CTTGTTCGTGTTGGGGATCTCTGGTTTGATGCAGCTTGTGTTCAATATAA

Consensus (1401) CTTGTTCGTGTTGGGGATCTCTGGTTTGATGCAGCTTGTGTTCAATATAA

1451 1500

Van-0 MOT1 (1397) TCCCTTTATCGGTTGTTAGAGGGATTCAGTTGTCACAAGGCTTAGCTTTT

Ler-0 MOT1 (1397) TCCCTTTATCGGTTGTTAGAGGGATTCAGTTGTCACAAGGCTTAGCTTTT

Col-0 MOT1 (1451) TCCCTTTATCGGTTGTTAGAGGGATTCAGTTGTCACAAGGCTTAGCTTTT

Consensus (1451) TCCCTTTATCGGTTGTTAGAGGGATTCAGTTGTCACAAGGCTTAGCTTTT

1501 1550

Van-0 MOT1 (1447) GCCATGTCTGCGGTTAAGTATATAAGGAAAGAGCAGAATTTTTTGAAGTC

Ler-0 MOT1 (1447) GCCATGTCTGCGGTTAAGTATATAAGGAAAGAGCAGAATTTTTCGAAGTC

Col-0 MOT1 (1501) GCCATGTCTGCGGTTAAGTATATAAGGAAAGAGCAGAATTTTTCGAAGTC

Consensus (1501) GCCATGTCTGCGGTTAAGTATATAAGGAAAGAGCAGAATTTTTCGAAGTC

1551 1600

Van-0 MOT1 (1497) AAAGAGTGTTGGTGATAGGCCATGGTTAGGGCTTGATGGTTTGGTTTTGG

Ler-0 MOT1 (1497) AAAGAGTGTTGGTGATAGGCCATGGTTAGGGCTTGATGGTTTGGTTTTGG

Col-0 MOT1 (1551) AAAGAGTGTTGGTGATAGGCCATGGTTAGGGCTTGATGGTTTGGTTTTGG

Consensus (1551) AAAGAGTGTTGGTGATAGGCCATGGTTAGGGCTTGATGGTTTGGTTTTGG

1601 1650

Van-0 MOT1 (1547) CTTTGGTTTGTGTTCTGTTCATAGTTCTTGTGAATGGAGATGGTGAAGAA

Ler-0 MOT1 (1547) CTTTGGTTTGTGTTCTGTTTATAGTTCTTGTGAATGGAGATGGTGAAGAA

Col-0 MOT1 (1601) CTTTGGTTTGTGTTCTGTTCATAGTTCTTGTGAATGGAGATGGTGAAGAA

Consensus (1601) CTTTGGTTTGTGTTCTGTTCATAGTTCTTGTGAATGGAGATGGTGAAGAA

1651 1700

Van-0 MOT1 (1597) GAAGAGGAAGAGGAAGAAGGAGATGGTTCGAGAGGAAGAGGAAGATGGGG

Ler-0 MOT1 (1597) GAAGAGGAAGAGGAAGAAGGAGATGGTTCGAGAGGAAGAGGAAGATGGGG

Col-0 MOT1 (1651) GAAGAGGAAGAGGAAGAAGGAGATGGTTCGAGAGGAAGAGGAAGATGGGG

Consensus (1651) GAAGAGGAAGAGGAAGAAGGAGATGGTTCGAGAGGAAGAGGAAGATGGGG

1701 1750

Van-0 MOT1 (1647) TTCGGTGAGGAAGGTTATAGCTAACGTGCCATCTGCTCTGTTGATATTCT

Ler-0 MOT1 (1647) TTCGGTGAGGAAGGTTATAGCTAACGTGCCATCTGCTCTGTTGATATTCT

Col-0 MOT1 (1701) TTCGGTGAGGAAGGTTATAGCTAACGTGCCATCTGCTCTGTTGATATTCT

Consensus (1701) TTCGGTGAGGAAGGTTATAGCTAACGTGCCATCTGCTCTGTTGATATTCT

1751 1800

Van-0 MOT1 (1697) TGTTGGGTGTTGTTTTGGCATTTATAAGGAAGCCGAGTATTGTACATGAC

Ler-0 MOT1 (1697) TGTTGGGTGTTGTTTTGGCATTTATAAGGAAGCCGAGTATTGTACATGAC

Col-0 MOT1 (1751) TGTTGGGTGTTGTTTTGGCATTTATAAGGAAGCCGAGTATTGTACATGAC

Consensus (1751) TGTTGGGTGTTGTTTTGGCATTTATAAGGAAGCCGAGTATTGTACATGAC

1801 1850

Van-0 MOT1 (1747) ATCAAGTTTGGACCGTCAAAGATGAAGATTGTGAGAATAAGCCGAAAAGC

Ler-0 MOT1 (1747) ATCAAGTTTGGACCGTCAAAGATGAAGATTGTGAGAATAAGCCGAAAAGC

Col-0 MOT1 (1801) ATCAAGTTTGGACCGTCAAAGATGAAGATTGTGAGAATAAGCCGAAAAGC

Consensus (1801) ATCAAGTTTGGACCGTCAAAGATGAAGATTGTGAGAATAAGCCGAAAAGC

1851 1900

Van-0 MOT1 (1797) ATGGAGAAACGGGTTTTTGAAAGGGACGGTCCCGCAGTTACCTCTTTCTG

Ler-0 MOT1 (1797) ATGGAGAAACGGGTTTTTGAAAGGGACGGTCCCGCAGTTACCTCTTTCTG

Col-0 MOT1 (1851) ATGGAGAAACGGGTTTTTGAAAGGGACGGTCCCGCAGTTACCTCTTTCTG

Consensus (1851) ATGGAGAAACGGGTTTTTGAAAGGGACGGTCCCGCAGTTACCTCTTTCTG

1901 1950

Van-0 MOT1 (1847) TTCTTAATTCTGTTGTGGCTGTGTGTAAGCTGTCGTATGATCTGTTCCCC

Ler-0 MOT1 (1847) TTCTTAATTCTGTTGTGGCTATGTGTAAGCTGTCGTATGATCTGTTCCCC

Col-0 MOT1 (1901) TTCTTAATTCTGTTGTGGCTGTGTGTAAGCTGTCGTATGATCTGTTCCCC

Consensus (1901) TTCTTAATTCTGTTGTGGCTGTGTGTAAGCTGTCGTATGATCTGTTCCCC

1951 2000

Van-0 MOT1 (1897) GAGAAGGAGTTCTCGGCTGCATCGGTTTCCATGACTGTTGGGCTGATGAA

Ler-0 MOT1 (1897) GAGAAGGAGTTCTCGGCTGCATCGGTTTCCATGACTGTTGGGCTGATGAA

Col-0 MOT1 (1951) GAGAAGGAGTTCTCGGCTGCATCGGTTTCCATGACTGTTGGGCTGATGAA

Consensus (1951) GAGAAGGAGTTCTCGGCTGCATCGGTTTCCATGACTGTTGGGCTGATGAA

2001 2050

Van-0 MOT1 (1947) TATGGTGGGATGTTGGTTTGGAGCAATGCCTACTTGTCATGGAGCTGGTG

Ler-0 MOT1 (1947) TATGGTGGGATGTTGGTTTGGAGCAATGCCTACTTGTCATGGAGCTGGTG

Col-0 MOT1 (2001) TATGGTGGGATGTTGGTTTGGAGCAATGCCTACTTGTCATGGAGCTGGTG

Consensus (2001) TATGGTGGGATGTTGGTTTGGAGCAATGCCTACTTGTCATGGAGCTGGTG

2051 2100

Van-0 MOT1 (1997) GTTTAGCCGGGCAGTATAAGTTTGGTGGGAGGAGTGGTGGGTGTGTGGCA

Ler-0 MOT1 (1997) GTTTAGCCGGGCAGTATAAGTTTGGTGGGAGGAGTGGTGGGTGTGTGGCA

Col-0 MOT1 (2051) GTTTAGCCGGGCAGTATAAGTTTGGTGGGAGGAGTGGTGGGTGTGTGGCA

Consensus (2051) GTTTAGCCGGGCAGTATAAGTTTGGTGGGAGGAGTGGTGGGTGTGTGGCA

2101 2150

Van-0 MOT1 (2047) CTGTTGGGAGTAGCTAAACTGGTGCTAGGGTTGGTCTTGGGAGGTTCATT

Ler-0 MOT1 (2047) CTGTTGGGAGTAGCTAAACTGGTGCTAGGGTTGGTCTTGGGAGGTTCATT

Col-0 MOT1 (2101) CTGTTGGGAGTAGCTAAACTGGTGCTAGGGTTGGTCTTGGGAGGTTCATT

Consensus (2101) CTGTTGGGAGTAGCTAAACTGGTGCTAGGGTTGGTCTTGGGAGGTTCATT

2151 2200

Van-0 MOT1 (2097) GGTGGGTATATTGGAGAAGTTTCCGGTTGGTGTGCTCGGGGCATTGCTAC

Ler-0 MOT1 (2097) GGTGGGTATATTGGAGAAGTTTCCGGTTGGTGTGCTCGGGGCATTGCTAC

Col-0 MOT1 (2151) GGTGGGTATATTGGAGAAGTTTCCGGTTGGTGTGCTCGGGGCATTGCTAC

Consensus (2151) GGTGGGTATATTGGAGAAGTTTCCGGTTGGTGTGCTCGGGGCATTGCTAC

2201 2250

Van-0 MOT1 (2147) TATTTGCAGGGGTAGAGCTTGCAATGGCGGCTAGAGATATGAATACAAAG

Ler-0 MOT1 (2147) TATTTGCAGGGGTAGAGCTTGCAATGGCGGCTAGAGATATGAATACAAAG

Col-0 MOT1 (2201) TATTTGCAGGGGTAGAGCTTGCAATGGCGGCTAGAGATATGAATACAAAG

Consensus (2201) TATTTGCAGGGGTAGAGCTTGCAATGGCGGCTAGAGATATGAATACAAAG

2251 2300

Van-0 MOT1 (2197) GGAGATGCATTTGTAATGCTTATGTGCACATCAGTCTCTTTGGGATCAAA

Ler-0 MOT1 (2197) GGAGATGCATTTGTAATGCTTATGTGCACATCAGTCTCTTTGGGATCAAA

Col-0 MOT1 (2251) GGAGATGCATTTGTAATGCTTATGTGCACATCAGTCTCTTTGGGATCAAA

Consensus (2251) GGAGATGCATTTGTAATGCTTATGTGCACATCAGTCTCTTTGGGATCAAA

2301 2350

Van-0 MOT1 (2247) TGCTGCCATAGGCTTTGTTGCTGGTGTTCTTTTGTATGTGGTTTTGTGGA

Ler-0 MOT1 (2247) TGCTGCCATAGGCTTTGTTGCTGGTGTTCTTTTGTATGTGGTTTTGTGGA

Col-0 MOT1 (2301) TGCTGCCATAGGCTTTGTTGCTGGTGATCTTTTGTATGTGGTTTTGTGGA

Consensus (2301) TGCTGCCATAGGCTTTGTTGCTGGTGTTCTTTTGTATGTGGTTTTGTGGA

2351 2400

Van-0 MOT1 (2297) TGCGGAACTACGGGCGAGCGAAGCCGAGCAGCCTTCCCCCGCAATCCGGT

Ler-0 MOT1 (2297) TGCGGAACTACGGGCGAGCGAAGCCGAGCAGCCTTCCCCCGCAATCCGGT

Col-0 MOT1 (2351) TGCGGAACTACGGGCGAGCGAAGCCGAGCAGCCTTCCCCCGCAATCCGGT

Consensus (2351) TGCGGAACTACGGGCGAGCGAAGCCGAGCAGCCTTCCCCCGCAATCCGGT

2401 2450

Van-0 MOT1 (2347) GAACATGCTTGAAAGTCTTCTTTTGTTGTGTGTAGTGTTCGCTCTTCTTC

Ler-0 MOT1 (2347) GAACATGCTTGAAAGTCTTCTTTTGTTGTGTGTAGTGTTCGCTCTTCTTC

Col-0 MOT1 (2401) GAACATGCTTGAAAGTCTTCTTTTGTTGTGTGTAGTGTTCGCTCTTCTTC

Consensus (2401) GAACATGCTTGAAAGTCTTCTTTTGTTGTGTGTAGTGTTCGCTCTTCTTC

2451 2500

Van-0 MOT1 (2397) TTAATGCTTTCGTATAAAAAGTTCAAAACTTCTTTGAACTGCATATCTAC

Ler-0 MOT1 (2397) TTAATGCTTTCGTATAAAAAGTTCAAAACTTCTTTGAACTGCATATCTAC

Col-0 MOT1 (2451) TTAATGCTTTCGTATAAAAAGTTCAAAACTTCTTTGAACTGCATATCTAC

Consensus (2451) TTAATGCTTTCGTATAAAAAGTTCAAAACTTCTTTGAACTGCATATCTAC

2501 2550

Van-0 MOT1 (2447) TCTGAATGAATTCAAATAAATCAGTGTACCATGTTTGTGATTACTTATTA

Ler-0 MOT1 (2447) TCTGAATGAATTCAAATAAATCAGTGTACCATGTTTGTGATTACTTATTA

Col-0 MOT1 (2501) TCTGAATGAATTCAAATAAATCAGTGTACCATGTTTGTGATTACTTATTA

Consensus (2501) TCTGAATGAATTCAAATAAATCAGTGTACCATGTTTGTGATTACTTATTA

2551 2600

Van-0 MOT1 (2497) ATAAAGAAAGTGAAGAGATGGACCAAATTGCTTTGACTGAAATCATCCGG

Ler-0 MOT1 (2497) ATAAAGAAAGTGAAGAGATGGACCAAATTGCTTTGACTGAAATCATCCGG

Col-0 MOT1 (2551) ATAAAGAAAGTGAAGAGATGGACCAAATTGCTTTGACTGAAATCATCCGG

Consensus (2551) ATAAAGAAAGTGAAGAGATGGACCAAATTGCTTTGACTGAAATCATCCGG

2601 2650

Van-0 MOT1 (2547) GGACTTTCAAAAACCATAGGGCAAAACAAACTGAAAAGAAATCCACCTAC

Ler-0 MOT1 (2547) GGACTTTCAAAAACCATAGGGCAAAACAAACTGAAAAGAAATCCACCTAC

Col-0 MOT1 (2601) GGACTTTCAAAAACCATAGGGCAAAACAAACTGAAAAGAAATCCACCTAC

Consensus (2601) GGACTTTCAAAAACCATAGGGCAAAACAAACTGAAAAGAAATCCACCTAC

2651

Van-0 MOT1 (2597) ATCTTCCC

Ler-0 MOT1 (2597) ATCTTCCC

Col-0 MOT1 (2651) ATCTTCCC

Consensus (2651) ATCTTCCC
