# Supplementary material for: Variation in Molybdenum Content Across Broadly Distributed Populations of Arabidopsis thaliana Is Controlled by a Mitochondrial Molybdenum Transporter (MOT1)
Source: PLoS Genet. 2008 Feb 29;4(2):e1000004. doi: 10.1371/journal.pgen.1000004 (PMC2265440; doi:10.1371/journal.pgen.1000004)
Supplement: Text S2 — Arabidopsis accessions used in the association mapping study. (0.03 MB DOC) [file pgen.1000004.s004.doc]

**Name ABRC ID**

An-1 CS22626

Bay-0 CS22633

Bil-5 CS22578

Bil-7 CS22579

Bor-1 CS22590

Bor-4 CS22591

Br-0 CS22628

Bur-0 CS22656

C24 CS22620

CIBC-17 CS22603

CIBC-5 CS22602

Col-0 CS22625

Ct-1 CS22639

Cvi-0 CS22614

Eden-1 CS22572

Eden-2 CS22573

Edi-0 CS22657

Ei-2 CS22616

Est-1 CS22629

Fab-2 CS22576

Fab-4 CS22577

Fei-0 CS22645

Ga-0 CS22634

Got-22 CS22609

Got-7 CS22608

Gy-0 CS22631

HR-10 CS22597

HR-5 CS22596

Kas-2 CS22638

Kin-0 CS22654

Knox-10 CS22566

Knox-18 CS22567

Kondara CS22651

Kz-1 CS22606

Kz-9 CS22607

Ler-1 CS22618

LL-0 CS22650

Lov-1 CS22574

Lov-5 CS22575

Lp2-2 CS22594

Lp2-6 CS22595

Lz-0 CS22615

Mr-0 CS22640

Mrk-0 CS22635

Ms-0 CS22655

Mt-0 CS22642

Mz-0 CS22636

N13 CS22621

Nd-1 CS22619

NFA-10 CS22599

NFA-8 CS22598

Nok-3 CS22643

Omo2-1 CS22584

Omo2-3 CS22585

Oy-0 CS22658

Pna-10 CS22571

Pna-17 CS22570

Pro-0 CS22649

Pu2-23 CS22593

Pu2-7 CS22592

Ra-0 CS22632

Ren-1 CS22610

Ren-11 CS22611

Rmx-A02 CS22568

RRS-10 CS22565

RRS-7 CS22564

Se-0 CS22646

Shahdara CS22652

Sorbo CS22653

Spr1-2 CS22582

Spr1-6 CS22583

Sq-1 CS22600

Sq-8 CS22601

Tamm-2 CS22604

Tamm-27 CS22605

Ts-1 CS22647

Ts-5 CS22648

Tsu-1 CS22641

Ull2-3 CS22587

Ull2-5 CS22586

Uod-1 CS22612

Uod-7 CS22613

Var2-1 CS22580

Var2-6 CS22581

Wa-1 CS22644

Wei-0 CS22622

Ws-0 CS22623

Ws-2 CS22659

Wt-5 CS22637

Yo-0 CS22624

Zdr-1 CS22588

Zdr-6 CS22589
