# Supplementary material for: Variation in Molybdenum Content Across Broadly Distributed Populations of Arabidopsis thaliana Is Controlled by a Mitochondrial Molybdenum Transporter (MOT1)
Source: PLoS Genet. 2008 Feb 29;4(2):e1000004. doi: 10.1371/journal.pgen.1000004 (PMC2265440; doi:10.1371/journal.pgen.1000004)
Supplement: Text S3 — Significant genotype vs shoot Mo associations. (0.02 MB DOC) [file pgen.1000004.s005.doc]

**Supplemental File 3.** Additional loci associated with shoot Mo content

**Chromosome Base Pair p-value**

5 3227256 6.74E-05

1 8682419 0.000113367

4 241345 0.000139009

4 15251327 0.000187937

1 19672910 0.000574584

1 6040552 0.000662418
